# Non-muscle myosin IIC predominantly expressed in the slow-twitch skeletal muscles impedes age-related muscle weakness

**DOI:** 10.1371/journal.pone.0337708

**Published:** 2025-12-04

**Authors:** Hiroki Hamaguchi, Shino Hiraoka, Kun Tang, Haonan Zhu, Yoshitaka Mita, Yasuro Furuichi, Nobuharu L. Fujii, Yasuko Manabe

**Affiliations:** 1 Takasaki Institute for Advanced Quantum Science, National Institutes for Quantum Science and Technology, Watanukimachi, Takasaki-shi, Gunma, Japan; 2 Department of Health Promotion Sciences, Graduate School of Human Health Sciences, Tokyo Metropolitan University, Minami-Osawa, Hachioji-shi, Tokyo, Japan; 3 Department of Immunology, Graduate School of Medicine, Juntendo University, Hongo, Bunkyo-ku, Tokyo, Japan; Fujita Health University: Fujita Ika Daigaku, JAPAN

## Abstract

Skeletal muscle expresses three types of non-muscle myosin (NM) II in addition to skeletal type myosin. While immature myoblasts have been reported to express NMIIA and NMIIB, playing roles in cell morphology, the specific localization and function of NMIIC in skeletal muscle cells remain unclear. In this study, we aimed to investigate the expression pattern and the physiological role of NMIIC in skeletal muscle. NMIIC was specifically expressed in the slow-twitch muscles such as soleus, which primarily consists of type I and type IIa fibers, and its expression increased as muscle differentiation progressed. To explore the function of NMIIC in skeletal muscle, we used whole-body NMIIC knockout (KO) mice. Myofiber size was slightly but significantly decreased in the soleus of young (18–20-week-old) NMIIC KO mice. However, contractile force of the isolated soleus muscle in the NMIIC KO mice did not differ from that of wild-type mice, suggesting that the slight reduction in fiber size has limited physiological significance at this age. Interestingly, in 81-week-old NMIIC KO mice, soleus contractile force was significantly reduced despite no difference in fiber size between aged wild-type and NMIIC KO mice. Notably, NMIIC expression levels were higher in aged than young mice. These findings suggest that while NMIIC has minimal impact on skeletal muscle function under young and healthy conditions, it may play a crucial role in maintaining muscle function when muscle is compromised at age.

## Introduction

The society is rapidly aging worldwide. The United Nations estimates that the number of individuals aged ≥ 80 years will triple between 2021 and 2050 [1], increasing the desire for healthy and independent lives, even among the elderly. Decline in muscle mass and contractile function with aging limits the overall movement, necessitates nursing care, and increases the risk of various diseases and mortality [2,3]. Therefore, maintaining muscle health, including muscle contractile and metabolic functions, is essential to prolong the life expectancy.

Skeletal muscle contraction occurs when thick myosin filaments interact with the thin actin filaments, facilitating movement and maintenance of posture. Herein, myosin refers to the class II myosin. Myosins are present in almost all eukaryotes, including plants and animals, and constitute a large and diverse superfamily [4]. Class II myosins expressed in animals are actin-based motor proteins grouped into skeletal, cardiac, and smooth muscle and non-muscle myosin II (NMII) with similar biological properties [5]. Skeletal muscles express NMII in addition to skeletal muscle myosin which is directly involved in muscle contraction as described above. NMII consists of a pair of heavy chains and two pairs of light chains (essential light chain and regulatory light chain [RLC]). The heavy chain consists of a globular head domain containing an ATP- and actin-binding site, neck domain, which is bound to the essential light chain and RLC, and tail rod domain [6]. Upon phosphorylation, RLC structure is changed to its active form, and it binds and cross-links actin filaments and exerts tension, playing crucial roles in cytokinesis [6], cell morphology [5,6], and migration [7,8].

Three NM isoforms (IIA, IIB, and IIC) encoded by three different genes (*Myh9*, *Myh10*, and *Myh14*, respectively) have been identified in mammals [7–9]. In skeletal muscles, NMIIA and NMIIB are expressed in the immature skeletal myoblasts and play distinct roles in cell morphology [10]. On the other hand, NMIIC is absent in myoblasts and has therefore been considered irrelevant to developmental processes; as a result, it has attracted limited attention. Indeed, whole-body NMIIC knockout (KO) mice develop normally and exhibit no apparent systemic abnormalities other than hearing impairment [11,12]. Nevertheless, NMIIC expression is markedly elevated in differentiated myotubes, suggesting that NMIIC plays a role in skeletal muscle physiology, although its precise function remains largely undefined.

Skeletal muscle tissues consist of myofibers, which are mature skeletal muscle cells. Myofibers are categorized into two types based on their myosin heavy chain (MyHC) patterns, contractile speed, fatigue resistance, and metabolic properties [13]. Slow-twitch (type I) fibers are characterized by MyHC I expression, high amount of mitochondria, oxidative capacity, and fatigue resistance, and low speed and power. In contrast, fast-twitch (type II) fibers are characterized by MyHC II expression, high glycolytic property, power, and speed, and low endurance. Fast-twitch fibers are further subcategorized into types IIa, IIx, and IIb based on the MyHC isoform and metabolic capacity. Types IIa, IIx, and IIb exhibit oxidative, intermediate, and glycolytic metabolic properties, respectively. Skeletal muscles are composed of a mixture of many muscle fibers; therefore, muscle tissue properties are determined by their fiber composition. For example, soleus is a slow-twitch muscle mainly consisting of type I and type IIa fibers, whereas extensor digitorum longus (EDL) is a fast-twitch muscle mainly consisting of type IIx and type IIb fibers [14,15]. Since muscle fibers indicate different protein expression patterns for each fiber type, determining which types of muscle fibers express NMIIC is important for understanding the physiological role of NMIIC in the skeletal muscle.

In this study, we aimed to determine the expression pattern of NMIIC in matured skeletal muscle cells and to examine the physiological significance of NMIIC in skeletal muscle functions. NMIIC was highly expressed in the type I and IIa fibers mainly present in the slow-twitch muscles. NMIIC KO mice showed no obvious phenotype at a young age. Notably, NMIIC deficiency in aged mice significantly decreased the contractile force in soleus, despite the fibers being comparable in size in soleus. Interestingly, the NMIIC expression was increased in aged mice with preserved muscle mass compared to young mice. Our results suggest that NMIIC has little effect on the skeletal muscle contractile function at a young age but plays an important role in maintaining the contractile force in muscles in old age.

## Materials and methods

### Ethics statement

All animal experiments were approved by the Animal Care Committee of Tokyo Metropolitan University (approval numbers: A2-16, A3-6, A4-3) and adhered to the relevant guidelines and regulations, including the Animal Research: Reporting of In Vivo Experiments guidelines.

### Animals

C57BL/6 young mice (8–16-week-old) and aged mice (82–86-week-old) were purchased from Charles River Laboratories Japan (Yokohama, Japan). Myh7-cyan fluorescent protein (CFP) mice (8–16-week-old) were purchased from The Jackson Laboratories (stock no. 016922; ME, USA). C57BL/6 aged mice and Myh7- CFP mice were housed in cages at 24 °C under a 12/12 h light/dark cycle. NMIIC KO mice were purchased from Cyagen (C57BL/6J-Myh14em1cyagen; serial number KOCMP-71960-Myh14-B6J; Cyagen Biosciences, CA, USA). The mice were generated via clustered regularly interspaced palindromic repeat (CRISPR)/CRISPR-associated protein 9 gene editing using a single guide RNA that deleted 10302 bp from exon 2 to exon 8 of *Myh14*. Young NMIIC KO mice (18–20 weeks old) and aged NMIIC KO mice (81 weeks old) were raised under the same conditions until the age at which they were used in the experiment. C57BL/6 aged mice, Myh7- CFP mice, and NMIIC KO mice were housed in cages at 24 °C under a 12/12 h light/dark cycle. The experimental period lasted up to 81 weeks, including both the animal housing and experimental phases. Due to the long-term nature of this study, which required animal housing for over one-year, humane endpoints were established to ensure animal welfare throughout the experimental period. Animals were monitored once in two days, and euthanasia was performed if any of the following criteria were met: body weight loss exceeding 20% of baseline, persistent self-injury or open wounds, severe lethargy or unresponsiveness, inability to access food or water, or signs of severe pain or distress. Once animals met the endpoint criteria, euthanasia was conducted within 1 h by trained personnel. Mice were euthanized either upon reaching humane endpoint criteria or when scheduled for skeletal muscle isolation. Euthanasia was performed via cervical dislocation under deep anesthesia using a three-agent cocktail (medetomidine [0.3 mg/kg body weight], midazolam [4 mg/kg body weight], and butorphanol tartrate [5 mg/kg body weight]), in accordance with approved protocols. In this study, a total of 82 mice were used and no animals died spontaneously before meeting the predefined endpoint criteria. All procedures were performed by personnel who had completed institutional training in laboratory animal handling, breeding, anesthesia, and euthanasia.

### Genotyping

DNA isolated using an ear punch biopsy of NMIIC KO mice was analyzed via PCR to determine the genotype. The following primers were used for the wild-type (WT): 5’-AGATACTAGGCTGACCGGTTCTC-3’ and 5’-TGGCCCTGTCTCAGTTTCTGAT-3’. The following primers were used for NMIIC KO 5’-AACCAGCCACATAAGTGATAGGAA-3’ and 5’-TGGCCCTGTCTCAGTTTCTGAT-3’. The expected PCR amplicon size was 738 bp in WT mice and 795 bp in NMIIC KO mice. The following primers were used for the housekeeping gene, *T cell receptor delta constant* (*TCRd*): 5’-CAAATGTTGCTTGTCTGGTG-3’ and 5’-GTCAGTCGAGTGCACAGTTT-3’. The expected PCR amplicon size was 200 bp.

### Muscle force measurement

Muscle force was measured as previously described [16]. Briefly, both ends of isolated soleus were tied with silk threads and mounted on an apparatus in a medium consisting of the Krebs- Ringer bicarbonate buffer with 2 mM sodium pyruvate for 20 min at 37 ºC. The muscle was stimulated with an electric pulse for 10 min under the following conditions: train rate = 1/min, train duration = 10 s, pulse rate = 100 Hz, pulse duration = 0.1 ms, and volts = 100 V (one train consists of 10 s of stimulation followed by 50 s of rest). Then, peak force, force generation, and integrated force generation were analyzed as the integrated values for each 10 s increment over a 10 min period using the PowerLab system (AD Instruments, Sydney, Australia) and corrected for soleus weight after electrical stimulation. Specifically, the peak force was analyzed as the maximum force generated during each train. The force generation was calculated as the integrated value for 10 s of each train. The integrated force generation was defined as the total force generated over 10 trains.

### Metabolic parameter measurement

To measure the metabolic parameters, 10-week-old mice were housed in a metabolic cage and acclimatized for 24 h at 24 °C under a 12/12 h light/dark cycle and respiratory quotient (RQ) and spontaneous physical activity were evaluated using the ARCO system (ARCO-2000 and Actracer-2000; Arcosystem, Chiba, Japan). Spontaneous physical activity was measured as the number of infrared light detection counts proportional to movement.

### Fiber isolation and primary cell culture

Skeletal muscle cells were cultured as previously described [17], with some modifications. Briefly, EDL and soleus muscles of WT, NMIIC KO, and Myh7-CFP mice were digested with 0.2% type I collagenase (Worthington, NJ, USA) and 1% antibiotic (Thermo Fisher Scientific, MA, USA) in high-glucose Dulbecco’s Modified Eagle’s Medium containing GlutaMAX (Thermo Fisher Scientific) at 37 °C for 2 h, followed by the isolation of single fibers under a stereo microscope (Leica Microsystems, Wetzlar, Germany). In Myh7-CFP mice, CFP fluorescence was observed under a fluorescence stereomicroscope (M165 FC; Leica Microsystems to identify the type I (CFP-positive) and type II (CFP-negative) fibers. The collected fibers were sonicated in a lysis buffer [18] and centrifuged at 13,000 ×* g* for 15 min at 4 °C. Then, protein concentration in the supernatant was determined via Bradford protein assay, followed by immunoblotting analysis.

Primary cells of isolated fibers were cultured as previously described [19]. Briefly, the cells were cultured on Matrigel (BD Biosciences, Franklin Lakes, NJ, USA)-coated dishes in a growth medium consisting of Dulbecco’s Modified Eagle’s Medium with no glucose (Thermo Fisher Scientific) supplemented with 30% fetal bovine serum (511−98,175; FUJIFILM Wako Pure Corporation Chemical, Osaka, Japan), 1% GlutaMAX (Thermo Fisher Scientific), 1% chicken embryo extract (US Biological, Marblehead, MA, USA), 10 ng/mL basic fibroblast growth factor (Thermo Fisher Scientific), and 1% antibiotic (Thermo Fisher Scientific) at 37 °C with 5% CO_2_. Myogenic differentiation was induced using a differentiation medium consisting of high-glucose Dulbecco’s Modified Eagle’s Medium supplemented with 5% horse serum (16050−130; Thermo Fisher Scientific) and 1% antibiotic. Subsequently, proteins were extracted from the cells as previously described [18].

### Evaluation of myoblast proliferation and differentiation

To evaluate myoblast proliferation, 20 myofibers of soleus were seeded into each well of a 24-well plate with the growth medium for six days. Myoblasts were fixed with 4% paraformaldehyde (PFA; FUJIFILM Wako Pure Corporation Chemical), and nuclei were stained with 4’,6-diamidino-2-phenylindole dihydrochloride. To evaluate myotube differentiation, three days after the initiation of differentiation, the myotubes were fixed with 4% PFA and blocked/permeabilized in phosphate-buffered saline (PBS) containing 0.3% Triton X-100 (Sigma-Aldrich, MO, USA) and 5% goat serum (Jackson Immuno Research Laboratories, PA, USA) for 30 min at room temperature. After incubation with MyHC antibodies (MAB4470; R&D Systems, MN, USA) at 4 °C overnight, the cells were cultured with the Alexa Fluor 488-conjugated secondary antibodies (Thermo Fisher Scientific) for 1 h, and nuclei were stained with 4’,6-diamidino-2-phenylindole dihydrochloride. The fluorescence was observed using a fluorescence microscope (BZ-X810; Keyence, Osaka, Japan), and the fusion index, the ratio of the number of nuclei in myotubes to the total number of nuclei, was calculated.

### Immunohistochemistry

Calf muscles of WT and NMIIC KO mice were embedded with the optimal cutting temperature compound (Sakura Finetek, Tokyo, Japan) and rapidly frozen. Then, muscle tissues were thinly sectioned at 10 µm with a cryostat (Leica), mounted on glass slides (Matsunami Glass, Osaka, Japan), fixed with 4% PFA for 10 min at room temperature, and treated with HistOne (Nacalai Tesque, Kyoto, Japan) at 70 °C for 20 min. The fixed slices were blocked with 5% goat serum dissolved in PBS containing 0.3% Triton-X for 30 min at room temperature and incubated with the primary antibodies against NMIIC (rabbit, monoclonal, 8189; Cell Signaling, MA, USA) and laminin-α2 (rabbit, polyclonal, L9393; Sigma-Aldrich) overnight at 4 °C. After washing with PBS containing 0.01% Triton-X, the slices were incubated with 594-conjugated secondary antibodies (Thermo Fisher Scientific) for 1 h at room temperature. To determine the muscle fiber type, the slices were incubated with primary antibodies against MyHC I (mouse, monoclonal, BA-F8; Developmental Studies Hybridoma Bank IA, USA), MyHC IIa (mouse, monoclonal, SC-71; Developmental Studies Hybridoma Bank), and MyHC IIb (mouse, monoclonal, BF-F3; Developmental Studies Hybridoma Bank) for 2 h at room temperature, followed by incubation with a mixture of secondary antibodies, including Alexa Fluor 350 IgG2b, Alexa Fluor 488 IgG1, and Alexa Fluor 555 IgM, for 1 h at room temperature. Fluorescence images of stained fibers were obtained using a fluorescence microscope (BZ-X810). Unstained fibers were identified as MyHC IIx. Fiber size was automatically analyzed for each fiber type and for all fibers combined in the cross section of the soleus based on fluorescence images, using the BZ-X800 analysis application (BZ-H4A; Keyence).

### Immunoblotting

Cell lysates were separated via sodium dodecyl sulfate-polyacrylamide gel electrophoresis and transferred onto polyvinylidene fluoride membranes. After blocking with Tris-buffered saline containing 0.1% Tween 20 and 5% bovine serum albumin and 5% non-fat dry milk or 2.5% non-fat dry milk and 2.5% bovine serum albumin, the membranes were incubated overnight with the anti-MyHC I (mouse, monoclonal, M8421; Sigma-Aldrich), anti-MyHC II (mouse, monoclonal, MABT840; Sigma-Aldrich), and anti-NMIIa (rabbit, polyclonal, 909801; BioLegend, SD, USA), anti-NMIIb (mouse, monoclonal, ab684; Abcam, Cambridge, UK), anti-NMIIC (rabbit, monoclonal, 8189; Cell Signaling), anti-cytochrome c oxidase (COX) IV (rabbit, polyclonal, 4844; Cell Signaling), anti-myoglobin (rabbit, monoclonal, ab77232; Abcam), anti-β-actin (rabbit, polyclonal, 4967; Cell Signaling), and anti-glyceraldehyde 3-phosphate dehydrogenase (GAPDH [rabbit, monoclonal, 2118; Cell Signaling]) primary antibodies. Then, horseradish peroxidase-conjugated anti-mouse IgG (GE Healthcare, Buckinghamshire, UK) or anti-mouse IgM mu chain (Abcam) secondary antibodies were used to detect chemiluminescence (PerkinElmer, Waltham, MA, USA).

### Evaluation of mitochondrial morphology in myofibers

To evaluate the mitochondrial morphology in myofibers, fibers of the soleus of WT and NMIIC KO mice were collected as described above. The fibers were stained with 50 nM MitoTracker Red CMXRos (M7512; Thermo Fisher Scientific) at 37 °C for 30 min, fixed with 4% PFA for 30 min, and observed under a confocal laser scanning microscope (FV-3000; EVIDENT, Tokyo, Japan). Finally, longitudinal length of mitochondria was analyzed using the ImageJ-Fiji software.

### Statistical analyses

Data are represented as the mean ± standard error of the mean. An unpaired Student’s *t*-test was used to evaluate the statistical differences between two groups. Variations in data taken at each time point were analyzed via one-way analysis of variance, followed by the Tukey-Kramer post-hoc test to compare the elapsed treatment time with the basal time. Statistical significance was set at *p *< 0.05.

## Results

### NMIIs expression levels at different stages of skeletal muscle cell development

**Fig 1:**
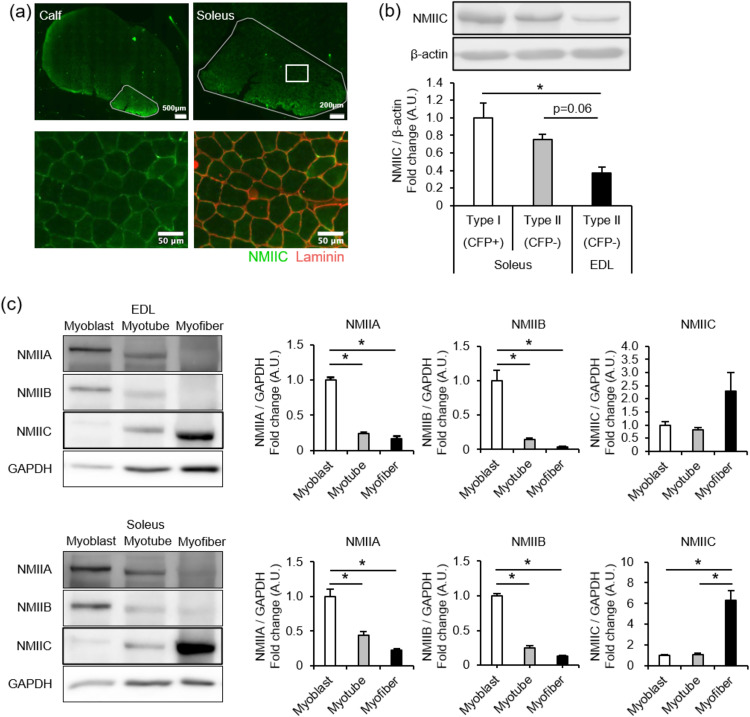
Expression of non-muscle myosin (NM) IIC in skeletal muscle cells. **(a)** NMIIC expression by immunohistochemistry. Upper: Whole calf (left; Scale bar, 500 μm) and soleus surrounded by a white line (right; Scale bar, 200 μm). Lower: Enlarged images of the white square area in the upper images. NMIIC and laminin were stained using antibodies against NMIIC (green) and laminin (red). Scale bar, 50 μm. **(b)** NMIIC expression levels in types I and II fibers of the soleus and extensor digitorum longus (EDL) muscles determined via immunoblotting. Types I (CFP+) and II (CFP-) fibers were collected separately from the soleus and EDL muscles of Myh7-cyan fluorescent protein (CFP) mice (see the Methods section). Expression was normalized to that of β-actin. Data are represented as the mean ± standard error of the mean (S.E.M.; n = 6; *p < 0.05 via one-way analysis of variance [ANOVA], followed by the Tukey-Kramer post-hoc test). *P*-values are 0.286, 0.003, and 0.063 for Types I vs. II of the soleus, Types I vs. II of the EDL, and Types II of the soleus vs. II of the EDL. **(c)** Representative image of immunoblotting and quantitative graph of NMIIA, NMIIB, and NMIIC expression levels in the myoblasts, myotubes, and myofibers of EDL and soleus. Expression was normalized to that of glyceraldehyde 3-phosphate dehydrogenase (GAPDH). Data are represented as the mean ± **S.**E.M. (n = 3; *p < 0.05 via one-way ANOVA, followed by the Tukey-Kramer post-hoc test). *P*-values of NMIIA from EDL-derived cells are < 0.001, < 0.001, and 0.35 for myoblasts vs. myotubes, myoblasts vs. myofibers, myotubes vs. myofibers, respectively. *P*-values of NMIIB from EDL-derived cells are 0.003, 0.002, and 0.768 for myoblasts vs. myotubes, myoblasts vs. myofibers, and myotubes vs. myofibers, respectively. *P*-values of NMIIC from EDL-derived cells are 0.962, 0.244, and 0.174 for myoblasts vs. myotubes, myoblasts vs. myofibers, and myotubes vs. myofibers, respectively. *P*-values of NMIIA from soleus-derived cells are 0.01, 0.002, and 0.307 for myoblasts vs. myotubes, myoblasts vs. myofibers, and myotubes vs. myofibers, respectively. *P*-values of NMIIB from soleus-derived cells are < 0.001, < 0.001, and 0.084 for myoblasts vs. myotubes, myoblasts vs. myofibers, and myotubes vs. myofibers, respectively. *P*-values of NMIIC from soleus-derived cells are 0.994, 0.003, and 0.003 for myoblasts vs. myotubes, myoblasts vs. myofibers, and myotubes vs. myofibers, respectively.

Calf muscles comprise the soleus (slow-twitch), gastrocnemius (fast-twitch), and plantaris (fast-twitch) muscles. To examine NMIIC distribution in skeletal muscle tissues, calf muscle sections were immunohistochemically stained with anti-NMIIC antibodies. As shown in Fig 1a, NMIIC was predominantly expressed in soleus and localized in the peripheral membranes. Soleus is mainly composed of type I fibers (slow twitch) and type IIa fibers (fast twitch with high oxidative capacity and fatigue resistance) [14]. To investigate whether NMIIC expression is distributed in a specific fiber type, 8–16-week-old Myh7-CFP mice, in which only type I fibers were labeled with CFP fluorescence, were used, as previously described [20]. In mice, type I (CFP+) and type II (CFP-) fibers from soleus were collected separately under a fluorescent microscope. Myofibers from EDL were collected as representative type IIb and IIx fibers. The collected fibers were homogenized and immunoblotted with anti-NMIIC antibodies. Consistent with the immunostaining results, NMIIC expression level was significantly higher in the type I (CFP+) fibers of soleus than in the type II (CFP-) fibers of EDL (Fig 1b). Within type II fibers, NMIIC expression level in the type II (CFP-) of soleus, mainly type IIa fibers, was higher than those in the type II (CFP-), mainly types IIx and IIb fibers of EDL. However, the difference was not significant. These results suggest that NMIIC is expressed mainly in the oxidative and fatigue-resistant type I and IIa fibers.

Next, NMIIC expression dynamics were investigated across cell differentiation lineages and compared with those of other isoforms, such as NMIIA and NMIIB expressed in the immature skeletal myoblasts [10]. During myogenesis, mononuclear myoblasts fuse to generate multinuclear myotubes that mature into myofibers. Immunoblotting was performed using primary cultures of myoblasts, myotubes, and myofibers isolated from EDL and soleus. Notably, NMIIA and NMIIB expression levels were higher in the myoblasts than in the myotubes and myofibers of EDL and soleus (Fig 1c). In contrast, the NMIIC expression level was significantly higher in the myofibers than in the myoblasts and myotubes of soleus (Fig 1c). NMIIC expression in EDL tended to be high in myofibers, but this failed to be statistically significant in cell differentiation lineages. The NMIIC band shown in Fig 1c was confirmed as correct using skeletal muscle tissue samples of NMIIC KO mice (described under ‘Phenotypic characteristics of NMIIC KO mice’) (S1a Fig). NMIIC expression was prominent in the mature skeletal muscle cells, and the expression patterns of NMIIC differed from those of NMIIA and NMIIB during myogenesis, suggesting that NMIIC plays distinct roles from NMIIA and NMIIB in skeletal muscle cells.

### Phenotypic characteristics of NMIIC KO mice

**Fig 2:**
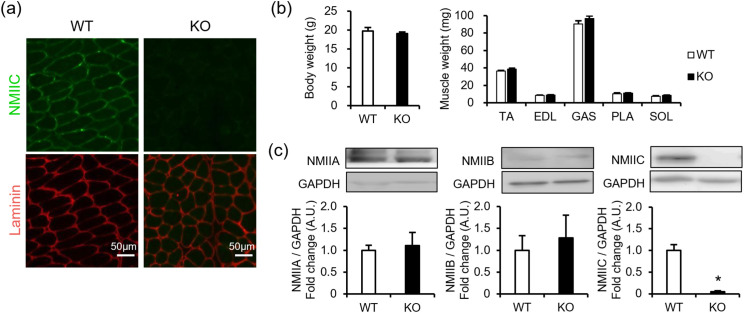
Phenotypic characterization of NMIIC knockout (KO) mice. **(a)** NMIIC expression by immunohistochemistry in the soleus of wild-type (WT) and NMIIC KO mice. Representative images are shown. Scale bar, 50 μm. **(b)** Body and muscle weights of WT and NMIIC KO mice. Eleven-week-old mice (n = 7) were used for body weight measurement, and 12-week-old mice (n = 6) were used for muscle weight measurement. Weights of tibialis anterior (TA), EDL, gastrocnemius (GAS), plantaris (PLA), and soleus (SOL) were measured. *P*-values from student’s *t*-test WT vs. KO are 0.583, 0.190, 0.467, 0.223, 0.387, and 0.389 for body, TA, EDL, GAS, PLA, and SOL weight, respectively. **(c)** NMIIA, NMIIB, and NMIIC expression levels in the soleus of WT and NMIIC KO mice. Expression was normalized to that of GAPDH. Data are represented as the mean ± **S.**E.M. (n = 4 [NMIIA and NMIIB] and n = 7-8 [NMIIC]). *p < 0.05 via Student’s *t*-*t*est. *P*-values from WT vs. KO are 0.785, 0.705, and <0.001 for NMIIA, NMIIB, and NMIIC, respectively.

Next, homozygous whole-body NMIIC KO mice were used to determine the specific roles of NMIIC in skeletal muscles (S1b Fig; Fig 2a). Although NMIIC protein expression was completely ablated in skeletal muscles, NMIIC KO mice grew normally, consistent with a previous report [12]. Body and skeletal muscle tissue weights of the tibialis anterior, EDL, gastrocnemius, plantaris, and soleus muscles were comparable between the WT and NMIIC KO mice (Fig 2b).

NMIIC deficiency is compensated by increased NMIIA and NMIIB expression levels in the organ of Corti [21]. To examine whether the expression levels of other NMII isoforms are increased in NMIIC KO mice, NMIIA and NMIIB expression levels in their soleus were measured. In contrast to a previous report [21], NMIIA and NMIIB expression levels in the soleus were comparable between the WT and NMIIC KO mice (Fig 2c). Therefore, NMIIC deficiency was not compensated by other NMII isoforms in skeletal muscle, suggesting that it plays different roles from NMIIA and NMIIB in mature skeletal muscle cells.

**Fig 3:**
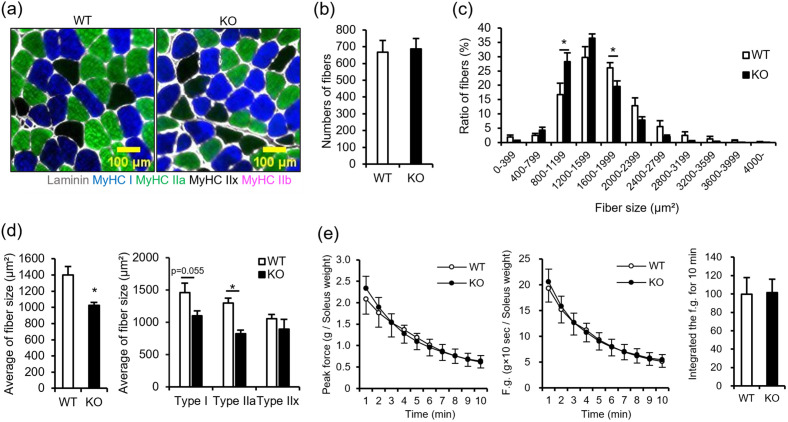
Fiber types and muscle force in the soleus of 18-20-week-old WT and NMIIC KO mice. **(a)** Myofiber type stains. Cross-sections of soleus were stained with the anti-MyHC I (blue; type I fiber), anti-MyHC IIa (green; type IIa fiber), and anti-MyHC IIb (red; type IIb fiber) antibodies. Unstained area (black; type IIx fiber) was assessed as the for MyHC IIx. Scale bar, 100 μm. **(b)** Average myofiber numbers in the soleus of WT and NMIIC KO mice. Data are represented as the mean ± **S.**E.M. (n = 7-8; Student’s *t*-test). *P*-value from WT vs. KO is 0.857. **(c)** Histogram of fiber size distribution in the soleus of WT and NMIIC KO mice. Data are represented as the mean ± **S.**E.M. (n = 7-8; Student’s *t*-test). *P*-values from WT vs. KO are 0.161, 0.182, 0.048, 0.165, 0.044, 0.118, 0.172, 0.202, 0.335, and 0.158 for 0-399, 400-799, 800-1199, 1200-1599, 1600-1999, 2000-2399, 2400-2799, 2800-3199, 3200-3599, 3600-3999, and 4000- μm^2^, respectively. **(d)** Cross-sectional area of each fiber type was quantified. (Left) Average area of all fiber types is shown. The average number of fibers used to analyze fiber size per condition were 667.6 ± 68.5 for WT mice and 685.9 ± 63.1 for NMIIC KO mice. (Right) Average area of each fiber type was shown. The average number of fibers used to analyze fiber size per condition in WT mice was 338.6 ± 37.5, 356.5 ± 35.2, and 22.3 ± 6.6 for Type I, Type IIa, and Type IIx, respectively. In NMIIC KO mice, the averages were 346.6 ± 37.5, 407.4 ± 50.8, and 8.9 ± 3.6, respectively. Data are represented as the mean ± **S.**E.M. (n = 6-8; *p < 0.05 via Student’s *t*-*t*est). *P*-values from WT vs. KO are 0.007, 0.055, < 0.001, and 0.842 for all fiber types, Type I fibers, Type IIa fibers, and Type IIx fibers, respectively. **(e)** Peak force and force generation stimulated by an electrical pulse in soleus. Isolated soleus was stimulated using an electric pulse for 10 min. One train consisted of 10 s stimulation (100 Hz; 0.1 ms duration; 100 V) and 50 s rest, which was repeated 10 times. (Left) Peak force is the maximum force exerted during 10 s/train corrected for soleus weight. (Middle) Force generation was calculated using the integrated values for 10 s of one train corrected for soleus weight. (Right) Integrated force generation was calculated for 10 min. Data are represented as the mean ± **S.**E.M. (n = 7-8; Student’s *t*-tes*t*). *P*-values WT vs. KO of peak force from are 0.575, 0.742, 0.994, 0.805, 0.788, 0.871, 0.947, 0.993, 0.958, and 0.902 for each train. *P*-values from WT vs. KO of peak generation are 0.740, 0.850, 0.999, 0.891, 0.925, 0.973, 0.966, 0.935, 0.912, and 0.838 for each train. *P*-value from WT vs. KO of integrated force generation is 0.936.

As NMIIC expression level was high in soleus (Fig 1b), we investigated whether NMIIC deficiency affects the myofiber size in the soleus of 18–20-week-old young mice. Notably, relatively smaller size of myofibers was increased in soleus of NMIIC KO, although total number of myofibers in soleus of NMIIC KO mice was comparable to that in WT (Fig 3a and 3b). A histogram of fiber size distribution revealed that the proportion of small-sized fibers was high and the average fiber size was significantly low in NMIIC KO mice. Type I fiber (slow twitch) and type IIa fiber (fast twitch with high oxidative capacity and fatigue resistance) size were particularly low in NMIIC KO mice (Fig 3c and 3d). To investigate whether the increased smaller size of myofibers in NMIIC KO mice affects muscle force generation, isolated soleus was stimulated using an electric pulse, and peak force, force generation, and integrated values over 10 s of one train for 10 min were calculated. As shown in Fig 3e, peak force, force generation, and integrated force generation after 10 min in NMIIC KO mice were not different from those in WT mice, suggesting that a small decrease in fiber size of NMIIC KO mice does not substantially affect the muscle contractile responses.

We also examined whether an increase in smaller size of fiber due to NMIIC deficiency might affect whole body activity and metabolic state. However, the locomotor activity and respiratory quotient (RQ), which fluctuates depending on the type of nutrients being metabolized, of NMIIC KO mice were comparable to that of WT mice (S2 Fig).

### NMIIC deficiency does not affect myoblast proliferation and differentiation into myotubes

**Fig 4:**
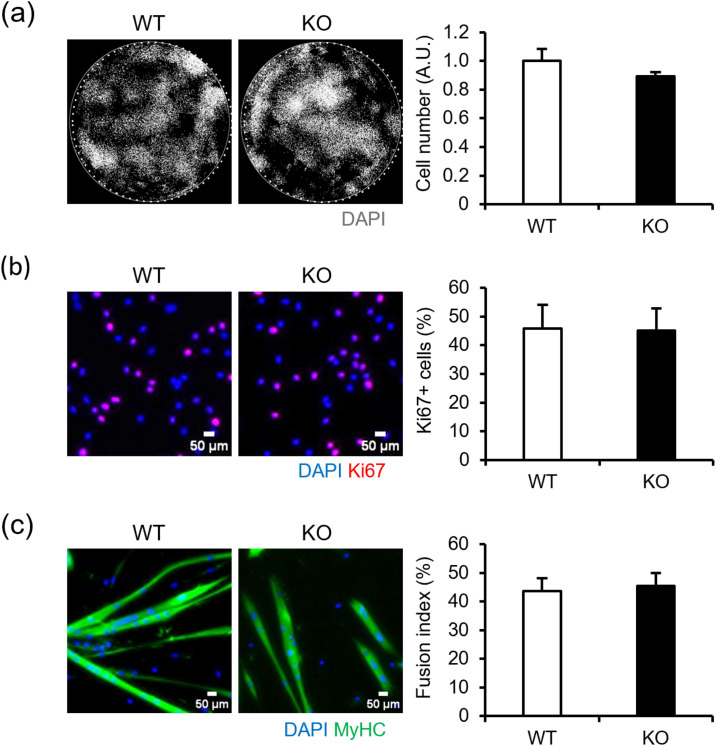
Proliferation and differentiation of myoblasts derived from satellite cells in WT and NMIIC KO mice. **(a)** Satellite cell proliferation. Satellite cells of 20 myofibers were seeded in 24-well plates. Numbers of cell nuclei stained with 4’,6-diamidino-2-phenylindole (DAPI) were measured. *P*-value from WT vs. KO is 0.344. **(b)** Proliferation ratio of myoblasts. Number of Ki67-positive cells (red) was determined and divided by the total cell number (cell nuclei stained with DAPI: blue). *P*-value from WT vs. KO is 0.965. **(c)** Fusion index of myotubes. Fusion index was calculated as the percentage of nuclei in myotubes stained with the anti-MyHC antibody (green) relative to the total number of nuclei (DAPI: blue). *P*-value from WT vs. KO is 0.794. Data are represented as the mean ± **S.**E.M. (n = 4- 6; Student’s *t*-test). Scale bar, 50 μm.

NMIIC was highly expressed in mature skeletal muscle cells, but its expression is also evident in immature myoblasts (Fig 1c). As NMIIA and NMIIB play key roles in immature myotubes [10], NMIIC may also be involved in the proliferation and differentiation of myoblasts during embryogenesis. To determine whether smaller size of myofibers is affected by abnormal cell proliferation and differentiation due to NMIIC deficiency, myoblast proliferation and myotube differentiation rates (fusion index) were calculated. However, both proliferation and differentiation rates were unaffected by NMIIC deficiency (Figs 4a-4c), suggesting that NMIIC does not impact myoblast proliferation and differentiation into myotubes.

### NMIIC deficiency affects the muscle force in aged mice

**Fig 5:**
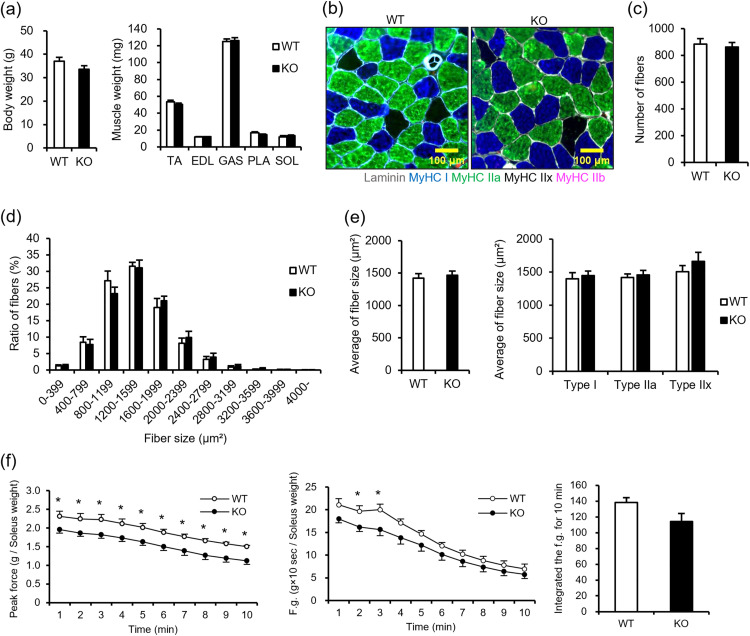
Phenotypic characterization of 81-week-old NMIIC KO mice. **(a)** Body and muscle weights of WT and NMIIC KO mice. Eighty-one-week-old mice were used. Data are represented as the mean ± **S.**E.M. (n = 5-7; Student’s *t*-test). *P*-values from WT vs. KO are 0.202, 0.152, 0.724, 0.941, 0.131, and 0.582 for body, TA, EDL, GAS, PLA, and SOL weight. **(b)** Myofiber type phenotyping stains. Cross-sections of the soleus muscle were stained with the anti-MyHC I (blue; type I fiber), anti-MyHC IIa (green; type IIa fiber), and anti-MyHC IIb (red; type IIb fiber) antibodies. Unstained area was assessed as the MyHC IIx (black; type IIx fiber). Scale bar, 100 μm. **(c)** Myofiber numbers in the soleus of WT and NMIIC KO mice. Numbers of fibers in the whole soleus area were counted. Data are represented as the mean ± **S.**E.M. (n = 5- 7; Student’s *t*-test). *P*-value from WT vs. KO is 0.720. **(d)** Histogram of fiber size distribution in the soleus of WT and NMIIC KO mice. Data are expressed as the percentage of the total fiber number. Data are represented as the mean ± **S.**E.M. (n = 5-7; Student’s *t*-test). *P*-values from WT vs. KO are 0.775, 0.782, 0.324, 0.852, 0.530, 0.551, 0.697, 0.635, 0.281, and 0.657 for 0-399, 400-799, 800-1199, 1200-1599, 1600-1999, 2000-2399, 2400-2799, 2800-3199, 3200-3599, 3600-3999, and 4000- μm^2^. **(e)** (Left) Average size of all fiber types in soleus. The average number of fibers used to analyze fiber size per condition was 884.6 ± 41.0 for WT mice and 862.7 ± 35.2 for NMIIC KO mice. (Right) Average fiber size of each fiber type in soleus. The average numbers of fibers used to analyze fiber size per condition in WT mice were 332.6 ± 15.1, 508.4 ± 39.8, and 43.6 ± 11.4 for Type I, Type IIa, and Type IIx, respectively. In NMIIC KO mice, the averages were 295.8 ± 16.4, 523.3 ± 19.3, and 43.9 ± 5.9 for those respective types, respectively. Data are represented as the mean ± **S.**E.M. (n = 5-7; Student’s *t*-test). *P*-values from WT vs. KO are 0.714, 0.712, 0.706, and 0.432 for all fiber types, Type I fibers, Type IIa fibers, and Type IIx fibers, respectively. **(f)** Peak force and force generation stimulated by an electrical pulse in the isolated soleus of WT and NMIIC KO mice. Isolated soleus was stimulated using an electric pulse for 10 min. One train consisted of 10 s stimulation (100 Hz; 0.1 ms duration; 100 V) and 50 s rest, which was repeated 10 times. (Left) Peak force is the maximum force exerted during 10 s/train corrected for soleus weight. (Middle) Force generation was calculated using the integrated values for 10 s of one train corrected for soleus weight. (Right) Integrated force generation was calculated for 10 min. Data are represented as the mean ± **S.**E.M. (n = 5-7). *p < 0.05 via Student’s *t*-*t*est. *P*-values WT vs. KO of peak force from are 0.047, 0.043, 0.030, 0.034, 0.023, 0.027, 0.029, 0.022, 0.016, and 0.011 for each train. *P*-values from WT vs. KO of peak generation are 0.078, 0.048, 0.049, 0.105, 0.184, 0.264, 0.328, 0.351, 0.364, and 0.398 for each train. *P*-value from WT vs. KO of integrated force generation is 0.102.

NMIIC deficiency affected the myofiber size in the soleus of young mice (Fig 3c-3d). However, this change did not affect the muscle force, suggesting that NMIIC is not critical for muscle functions at a young age. Subsequently, we investigated whether NMIIC deficiency affects the aged mice. Eighty-one-week-old mice were used as aged mice. Body weight, muscle weight, number of myofibers, and fiber size were not significantly different between the WT and NMIIC KO mice (Fig 5a-5e). Notably, peak force and force generation were significantly decreased in the isolated soleus of aged NMIIC KO mice (Fig 5f). Integrated force generation for 10 min was tended to decrease in the isolated soleus of aged NMIIC KO mice (Fig 5f).

### NMIIC deficiency does not affect the mitochondrial functions

**Fig 6:**
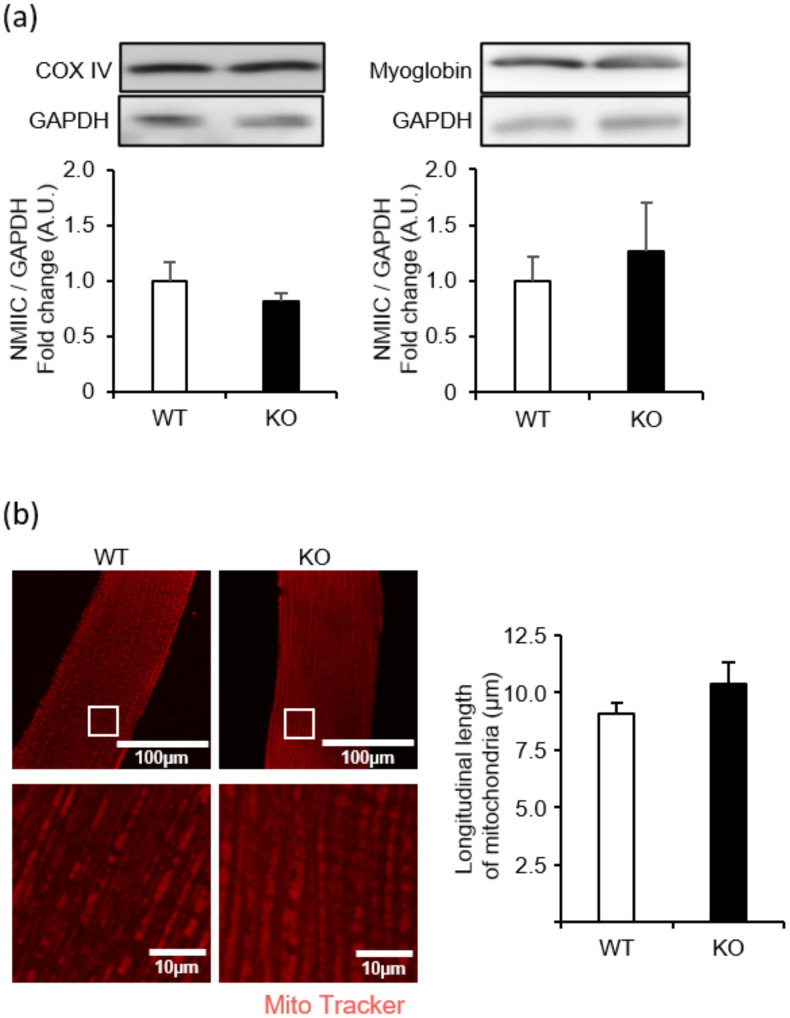
Evaluation of mitochondrial protein expression and morphology in NMIIC KO mice. **(a)** Cytochrome c oxidase (COX) IV and myoglobin expression levels in 81-week-old NMIIC KO mice. Representative image of immunoblotting and quantitative graphs of NMIIC levels. Expression was normalized to that of GAPDH. Data are represented as the mean ± **S.**E.M. (n = 5-7; Student’s *t*-test). *P*-values from WT vs. KO are 0.317 and 0.662 for COX IV and myoglobin, respectively. **(b)** Representative images of stained mitochondria in soleus fibers and longitudinal length of mitochondria. Upper image: Myofibers stained with Mito Tracker and observed via confocal fluorescence microscopy. Scale bar, 100 μm. Lower image: Enlarged image of the white square area of the upper image. Scale bar, 10 μm. Graph shows the average longitudinal length of mitochondria. Data are represented as the mean ± **S.**E.M. (n = 4; Student’s *t*-test).

Mutated NMIIC induces impaired mitochondrial fission, which is associated with peripheral neuropathy [22]. To determine whether NMIIC deficiency affects mitochondrial function, we evaluated the expression levels of the representative proteins related to mitochondrial function. Specifically, we examined the expression levels of COX IV as a representative mitochondrial protein and myoglobin as a heme protein supplying oxygen to mitochondria in NMIIC KO mice. COX IV and myoglobin expression levels were unaltered in the soleus of NMIIC KO mice (Fig 6a). Subsequently, we assessed the mitochondrial morphology in the soleus myofibers of NMIIC KO mice. No significant change in the longitudinal length of mitochondria was observed in NMIIC KO mice (Fig 6b), suggesting that mitochondrial fission is not affected in these mice.

### NMIIC expression in aged mice

**Fig 7:**
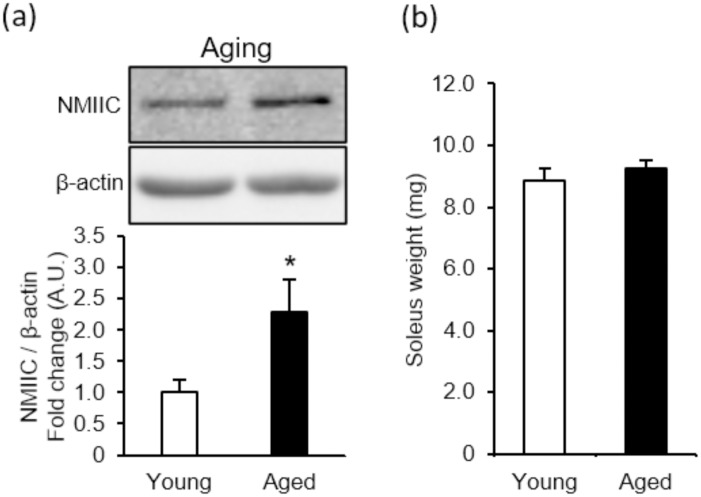
NMIIC expression and muscle weight in the soleus of 82-86-week-old mice. **(a)** Representative images of immunoblotting and quantitative graphs of NMIIC expression level. Expression was normalized to that of β-actin. *P*-values from Young vs. Aged is 0.047. **(b)** Muscle weight of the soleus in young (8-10-week-old) and aged (82-86-week-old) mice. *P*-values from Young vs. Aged is 0.405. Data are represented as the mean ± **S.**E.M. (n = 5-8). *p < 0.05 via Student’s *t*-*t*est.

In young mice, no significant differences in muscle force were observed between WT and NMIIC KO mice. In contrast, whereas aged WT mice maintained muscle force comparable to that of young WT mice (Fig 3e vs. Fig 5f; direct comparisons were not possible because measurements were performed on different days), aged NMIIC KO mice exhibited a marked reduction in muscle force. These results indicate that NMIIC plays a critical role in the preservation of muscle force in aged mice. To further investigate age-associated changes, we compared NMIIC expression between young and aged WT mice and found that NMIIC levels were significantly elevated in aged mice (Fig 7a). Generally, muscle weight decreases with aging in humans, and muscle loss with aging is smaller in mice than in humans and occurs relatively later in life [23]. Consistently, aged mice did not show any decrease in muscle weight in this study (Fig 7b). These findings suggest that upregulated NMIIC expression, particularly before muscle mass loss, may contribute to the preservation of muscle force during aging.

## Discussion

This study aimed to investigate the expression profile of NMIIC in matured skeletal muscle cells and to clarify its physiological significance in skeletal muscle function. We found that NMIIC expression was especially high in slow-twitch muscles such as the soleus, which consists mainly of type I and IIa fibers. NMIIC KO mice showed normal growth rates and no obvious phenotypes at a young age. Meanwhile, in 81-week-old mice, muscle contractile force in the soleus of NMIIC KO mice was significantly decreased. NMIIC expression level in aged mice before the loss of muscle mass occurred was significantly increased compared to that in young mice. Our results highlight the importance of NMIIC expression in maintaining the contractile force with gradually increasing muscle weakness in old age.

NMIIC expression level was high in differentiated myotubes and myofibers. Whereas NMIIA and NMIIB expression levels were observed in myoblast and myotubes but not in myofibers (Fig 1c). A previous study suggested that NMIIC deficiency is compensated by increased NMIIA and IIB expression levels [21]. However, NMIIA and NMIIB expression levels were not altered in the NMIIC KO mice in this study (Fig 2c). Expression patterns of NMIIC during development were different from those of NMIIA and NMIIB (Fig 1c), suggesting that it plays roles distinct from those of other NMII isoforms in skeletal muscles.

Here, the type I and IIa fibers of NMIIC KO mice at a young age were smaller in size, although the force generation, motor activity, and RQ were not affected (Figs 3d and 3e; S2 Fig). Optimal myoblast proliferation and differentiation are important for skeletal muscle development during embryogenesis. The NMIIC KO mice used in this study lack NMIIC during development, which may have initially affected the proliferation and differentiation of skeletal muscle cells, causing the increase in smaller fibers. However, NMIIC deficiency did not affect the proliferation and differentiation of skeletal muscle cells (Fig 4a-4c), suggesting that NMIIC is not associated with myofiber size in the early developmental stages. The reason for the increase in smaller fibers in the soleus muscle of NMIIC KO mice at a young age is unclear, but this change is considered only trivial at least at a young age, because it did not significantly affect muscle development, contractile function, and whole-body activity and metabolic state.

We previously reported that smaller myofiber size and reduced fiber number in Musashi-2 knockout mice were associated with decreased soleus contractile force [24]. In contrast, young NMIIC KO mice maintained muscle strength comparable to WT mice despite a modest reduction in fiber size (Fig 3d and 3e). The mechanism underlying this preserved force remains unclear; however, unlike Musashi-2 knockout mice, young NMIIC KO mice exhibited only a slight decrease in fiber size without changes in muscle mass or fiber number (Figs 2b and 3b), which may have minimized the impact on strength. In contrast, aged NMIIC KO mice showed a significant decline in muscle strength compared with aged WT mice, despite no detectable changes in fiber size or number, suggesting that NMIIC contributes to the maintenance of muscle strength in advanced age (Fig 5c, 5e, and 5f).

In contrast to young NMIIC KO mice, muscle contractile force in aged NMIIC KO mice was significantly decreased without any change in muscle weight and fiber size (Fig 5a-5e). NMIIC mutants reportedly trigger mitochondrial dysfunction and are associated with peripheral neuropathy [22]. As slow muscle fibers exhibit high mitochondrial content and oxidative capacity, the decreased soleus contractile force in old NMIIC KO mice was possibly due to mitochondrial dysfunction. Therefore, we determined the expression levels of COX IV as a representative mitochondrial protein and myoglobin as an oxygen-binding heme protein in NMIIC KO mice. However, COX IV and myoglobin expression levels were not altered in the soleus of NMIIC KO mice (Fig 6a). We also assessed the mitochondrial morphology in the soleus myofibers of NMIIC KO mice. No significant changes in the longitudinal length of mitochondria were observed (Fig 6b), suggesting that mitochondrial morphology is not impaired in NMIIC KO mice. Therefore, decreased muscle contractile force in aged NMIIC KO mice was not due to mitochondrial function indicators that we evaluated. To elucidate the mechanism underlying the decreased muscle contractile force in that, it may be necessary to evaluate other proteins involved in mitochondrial dysfunction and mitochondrial localization.

At present, specific mechanisms responsible for the significant differences in muscle strength remain unknown. These differences occur despite the absence of changes in soleus muscle weight in both young and aged mice and without the increase in small fibers seen in young NMIIC KO mice, an effect not observed in aged mice (S3a and S3b Fig). Loss of muscle strength is faster than the loss of muscle weight with aging in humans [25]. Although direct comparisons among species are difficult, Ballak et al. calculated the relative age of each species as a percentage based on the average lifespan (set at 100%) [23]. Based on this calculation, they revealed that the age-related loss of muscle mass in mice is much lower than that in humans and that mice begin to lose muscle mass at approximately 90% of their relative age (24-month-old). Here, we used mice aged 81 weeks (20 months), which is a borderline age for muscle atrophy onset. Therefore, the mice in this study would have only shown the muscle weakness.

With aging, skeletal muscle functions are affected by many factors, including a decline in muscle mass due to reduced protein synthesis and increased proteolysis [26], decline in motor neurons and neuromuscular junction dysfunction [27], and changes in fiber distribution [28]. These factors are retained at young age, which may compensate for NMIIC deficits and maintain muscle function at young age. However, these factors change and decline with aging, and may be unable to compensate for NMIIC deficiency, leading to a deterioration of muscle function. The increased NMIIC expression observed in soleus tissue of aged mice compared with young mice (Fig 7a) might be a physiological response to resist age-related muscle weakness occurring without changes in muscle mass or fiber size. Determining the stage of differentiation—myoblast, myotube, or myofiber—at which the age-related increase in NMIIC expression occurs remains a challenge, as isolation of single myofibers from aged mice proved too difficult.

One limitation of this study is that the specific roles of NMIIC in slow-twitch fibers were not elucidated. NMIIC contributes to tension homeostasis by forming a transcellular contractile sarcomeric network in epithelial cells [29], possibly contributing to tension homeostasis in skeletal muscle cells, thereby affecting the muscle contractility. Furthermore, NMIIC has three splicing variants [9,30]. In this study, we were unable to distinguish these variants by immunoblotting (Fig 1c and S1a Fig). Additionally, NMIIC KO mice lack all variants. Therefore, it remains unclear which variant increases and functions during aging. Future studies should investigate the association between the expression of NMIIC splicing variants and muscle strength in old patients for further insights.

In summary, NMIIC was mainly expressed in the slow-twitch muscles composed of type I and type IIa fibers. NMIIC deficiency decreased the muscle fiber size, but not the muscle functions, in young mice and muscle contractile force in aged mice. Overall, our results suggest that NMIIC has little effect on skeletal muscle functions at a young age but plays an important role in maintaining the muscle contractile force in old age.

## Supporting information

S1 Fig Confirmation of NMIIC expression. (a) Representative image of immunoblotting of NMIIC expression in the myoblasts, myotubes, myofibers, and tissues of soleus and EDL. Myoblasts, myotubes, and myofibers were derived from soleus and EDL muscles of WT mice. Tissues were harvested from NMIIC KO mice. Arrows indicate the NMIIC bands. (b) Confirmation of NMIIC deletion in NMIIC KO mice using PCR. *MYH14* gene coding for NMIIC KO was confirmed via PCR. The expected PCR amplicon size was 738 bp in WT mice and 795 bp in NMIIC KO mice. *TCRd* was used as a housekeeping gene.
(TIF)

S2 Fig Spontaneous physical activity and respiratory quotient (RQ) in WT and NMIIC KO mice. Accumulated physical activity over 24 h is expressed as the number of infrared light detection counts. RQ is expressed as an average over 24 h. Ten-week-old mice were used in this study. Data are represented as the mean ± standard error of the mean (S.E.M.; n = 5–7; Student’s *t*-test). *P*-values from WT vs. KO are 0.815 and 0.879 for spontaneous physical activity and respiratory quotient (RQ).
(TIF)

S3 Fig Comparison of soleus muscle weight and fiber size histogram between young and aged WT and NMIIC KO mice. Integrated data of soleus muscle weights and fiber size histogram in young (Fig 3) and aged (Fig 5) WT and NMIIC KO mice. (a) Soleus muscle weights of young and aged WT and NMIIC KO mice. (b) Fiber size histogram in the soleus of young and aged WT and NMIIC KO mice. Data are expressed as percentages of the total number of fibers. Data are represented as the mean ± S.E.M. (n = 5–8).
(TIF)

S1 Raw Images Original images for blots and gel. (PDF)

S1 Data Raw data for all figures and supporting information. (XLSX)

## Abbreviation:

CFP: cyan fluorescent protein
EDL: extensor digitorum longus
MyHC: myosin heavy chain
NM: non-muscle myosin
PBS: phosphate-buffered saline
PFA: paraformaldehyde
RLC: regulatory light chain
RQ: respiratory quotient
