# Supplementary figures and images for: Non-muscle myosin IIC predominantly expressed in the slow-twitch skeletal muscles impedes age-related muscle weakness
Source: PLoS One. 2025 Dec 4;20(12):e0337708. doi: 10.1371/journal.pone.0337708 (PMC12677458; doi:10.1371/journal.pone.0337708)

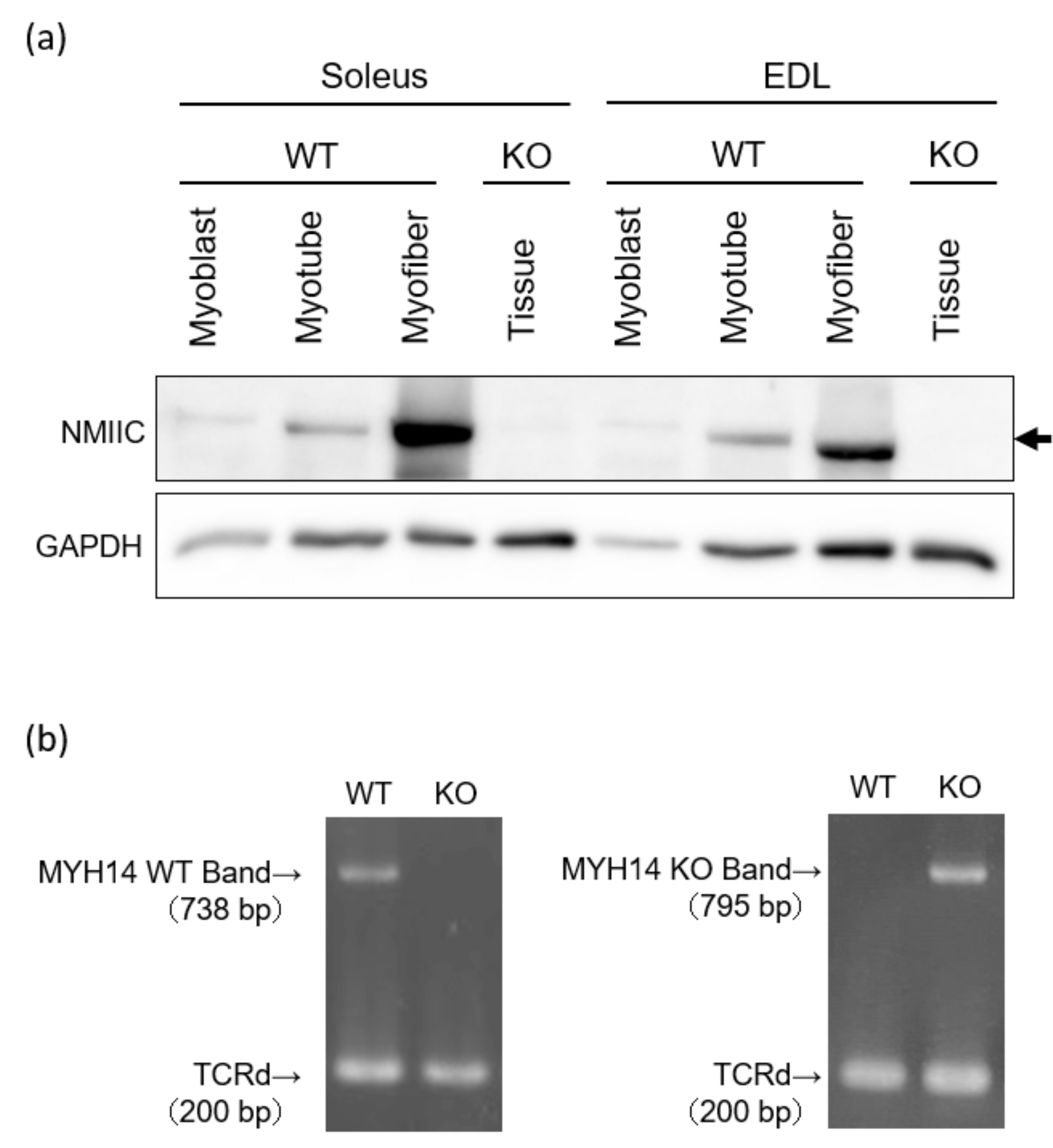

Supplement: S1 Fig — (a) Representative image of immunoblotting of NMIIC expression in the myoblasts, myotubes, myofibers, and tissues of soleus and EDL. Myoblasts, myotubes, and myofibers were derived from soleus and EDL muscles of WT mice. Tissues were harvested from NMIIC KO mice. Arrows indicate the NMIIC bands. (b) Confirmation of NMIIC deletion in NMIIC KO mice using PCR. MYH14 gene coding for NMIIC KO was confirmed via PCR. The expected PCR amplicon size was 738 bp in WT mice and 795 bp in NMIIC KO mice. TCRd was used as a housekeeping gene. (TIF) [file pone.0337708.s001.tif]

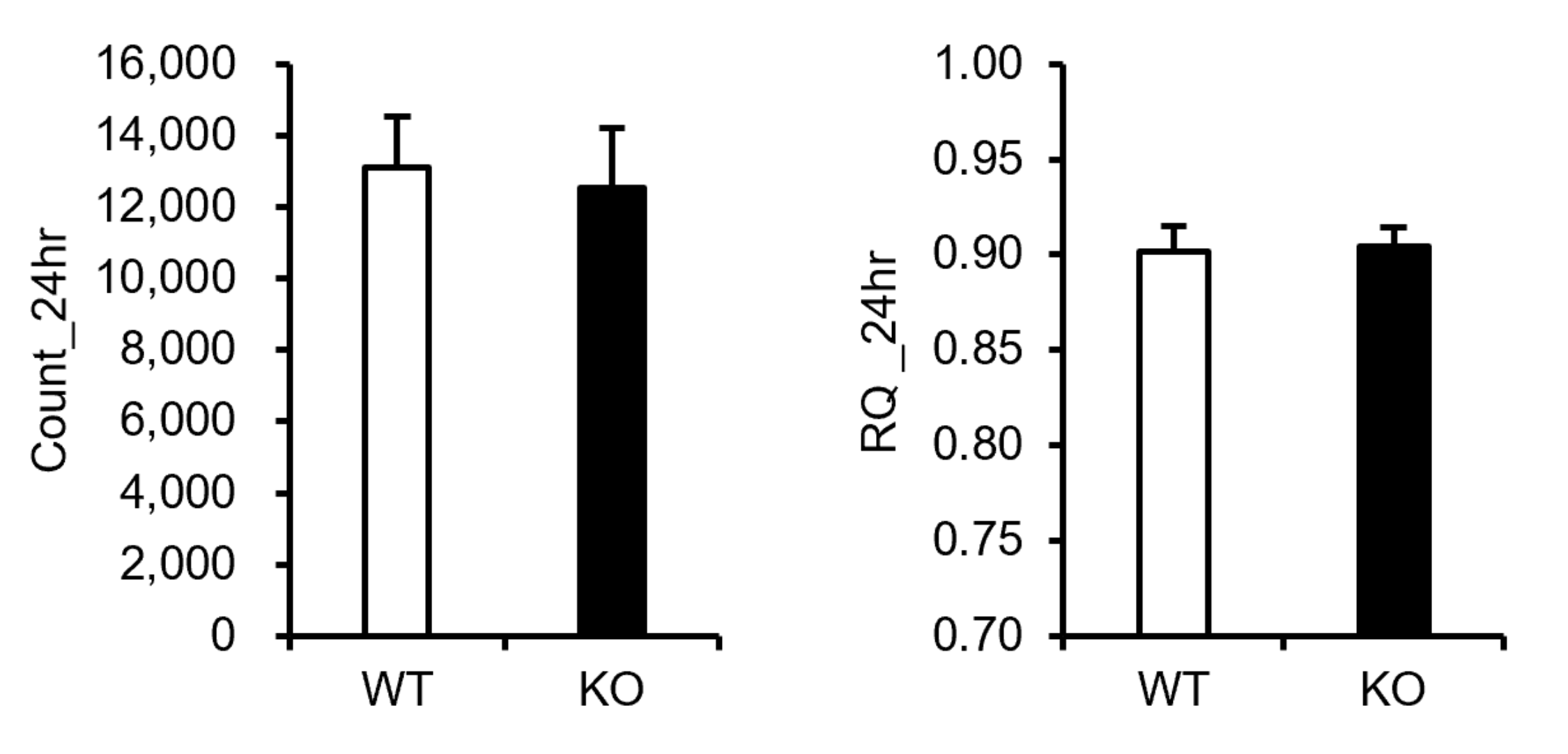

Supplement: S2 Fig — Accumulated physical activity over 24 h is expressed as the number of infrared light detection counts. RQ is expressed as an average over 24 h. Ten-week-old mice were used in this study. Data are represented as the mean ± standard error of the mean (S.E.M.; n = 5–7; Student’s t-test). P-values from WT vs. KO are 0.815 and 0.879 for spontaneous physical activity and respiratory quotient (RQ). (TIF) [file pone.0337708.s002.tif]

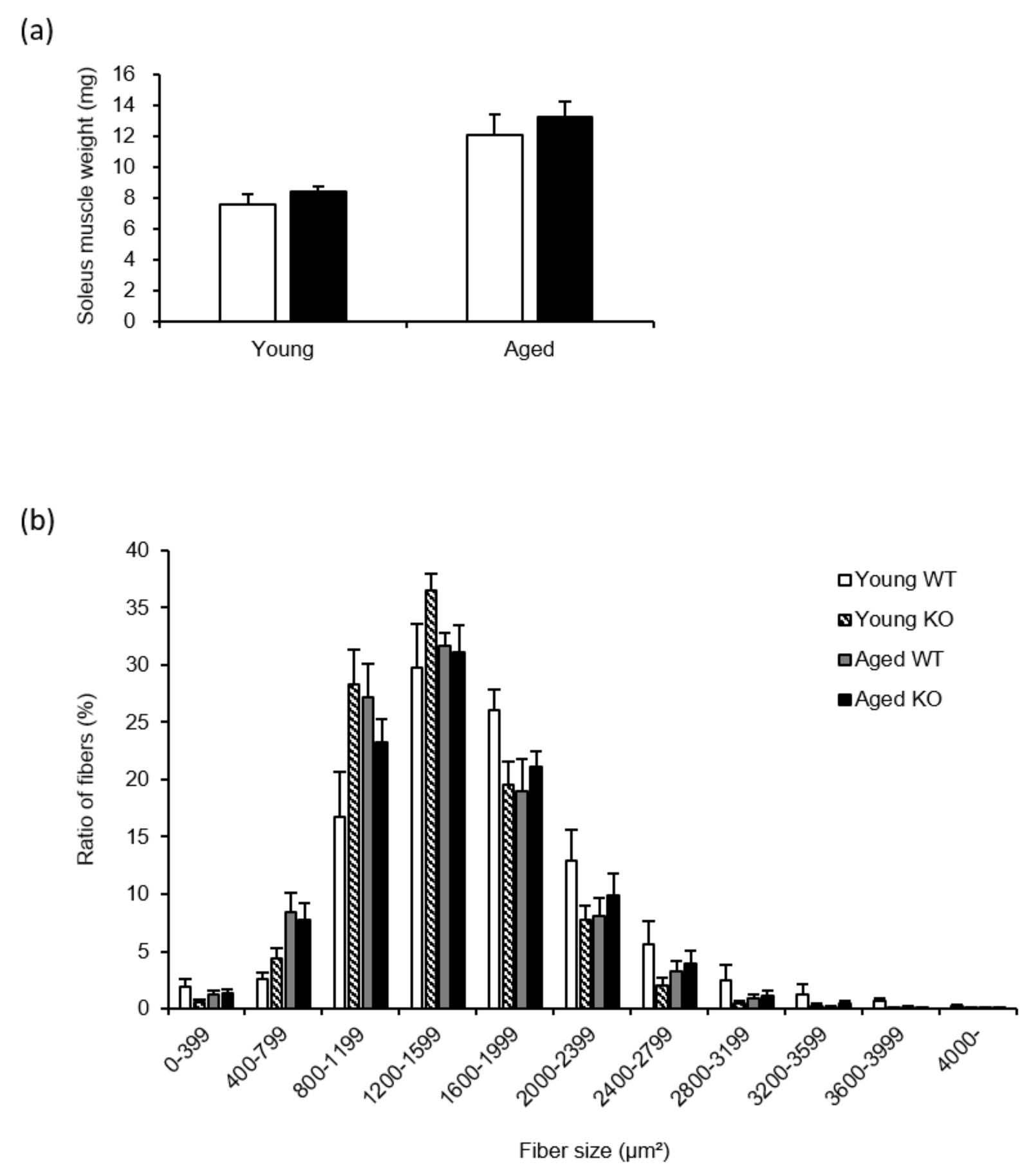

Supplement: S3 Fig — Integrated data of soleus muscle weights and fiber size histogram in young (Fig 3) and aged (Fig 5) WT and NMIIC KO mice. (a) Soleus muscle weights of young and aged WT and NMIIC KO mice. (b) Fiber size histogram in the soleus of young and aged WT and NMIIC KO mice. Data are expressed as percentages of the total number of fibers. Data are represented as the mean ± S.E.M. (n = 5–8). (TIF) [file pone.0337708.s003.tif]

Fig 1b

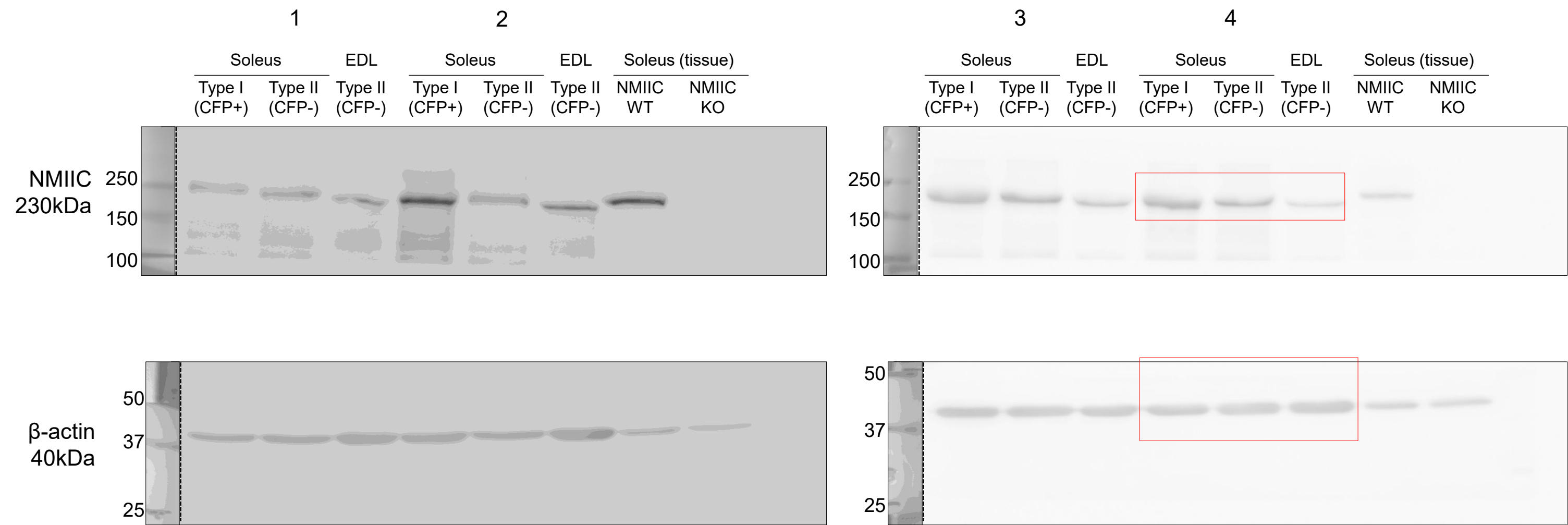

Fig 1c

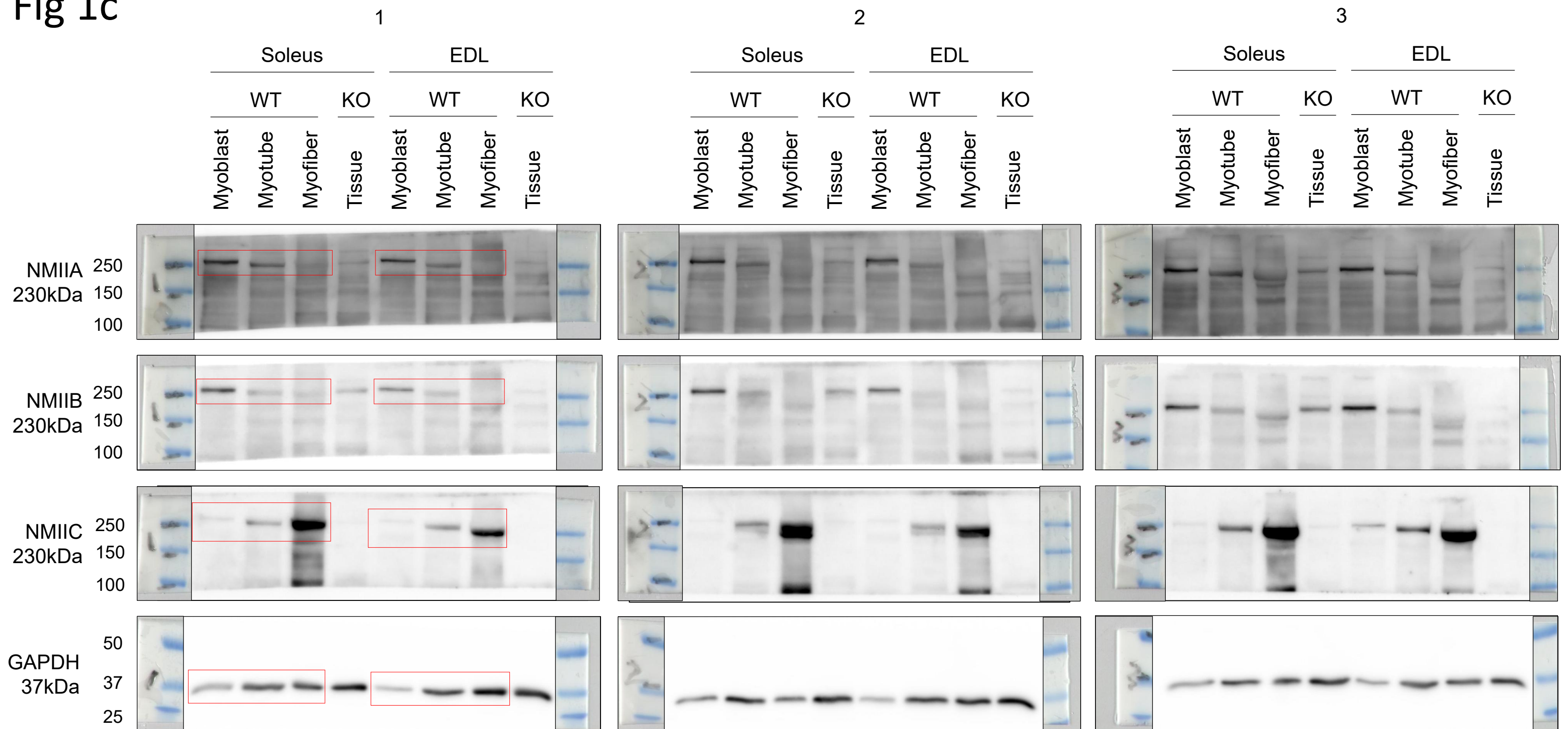

WT: Wild-type Mice  
KO: NMIIC KO Mice

Fig 2c

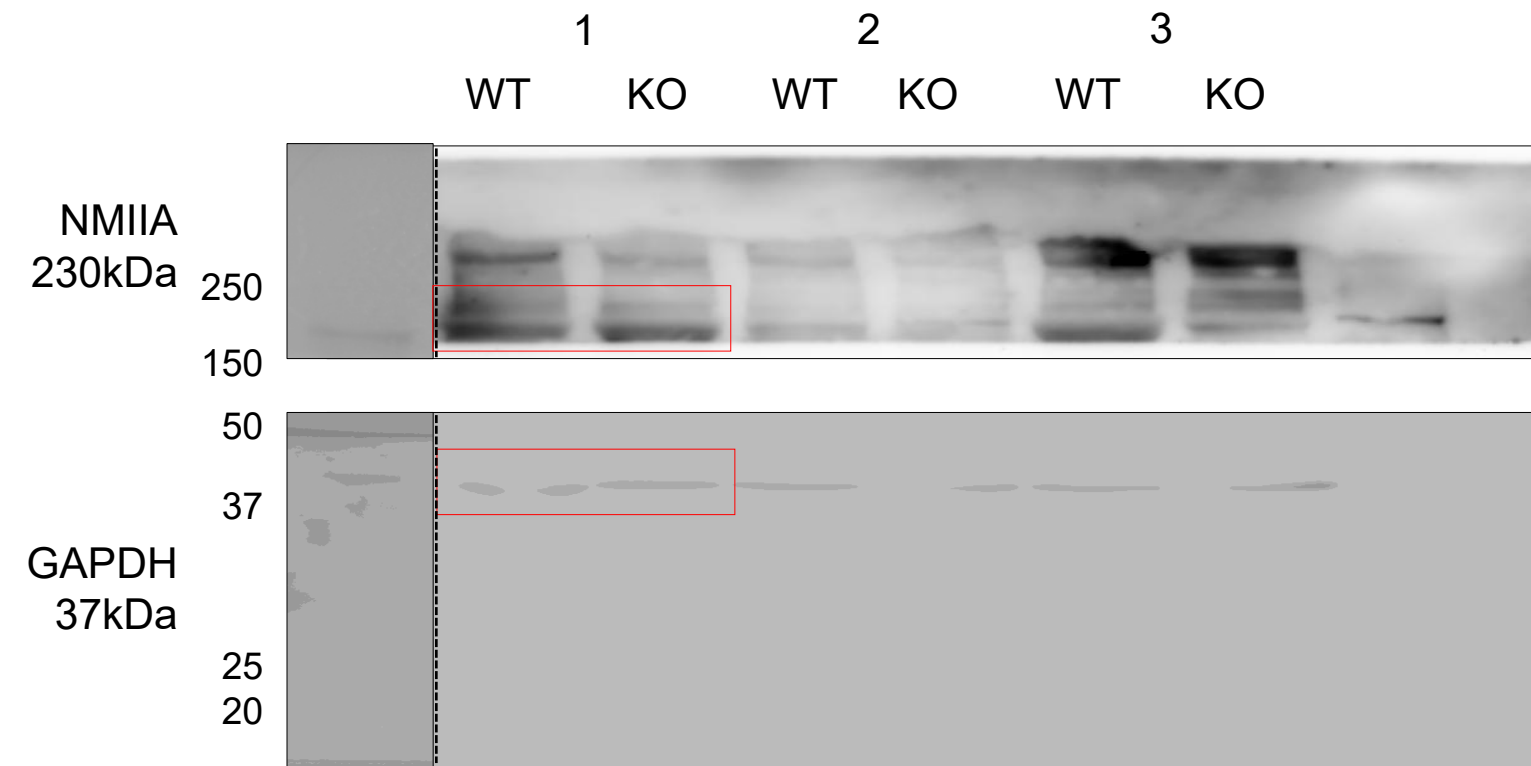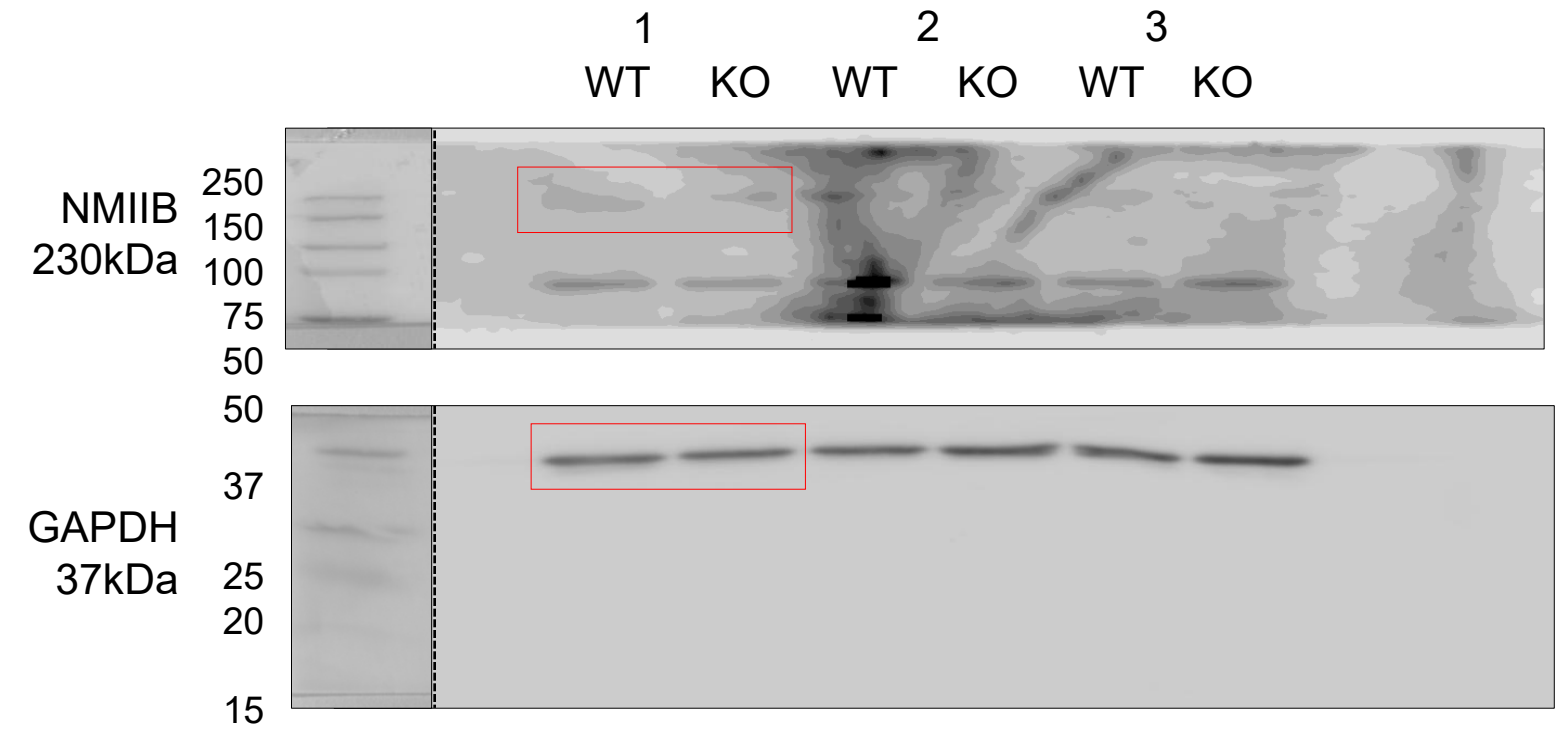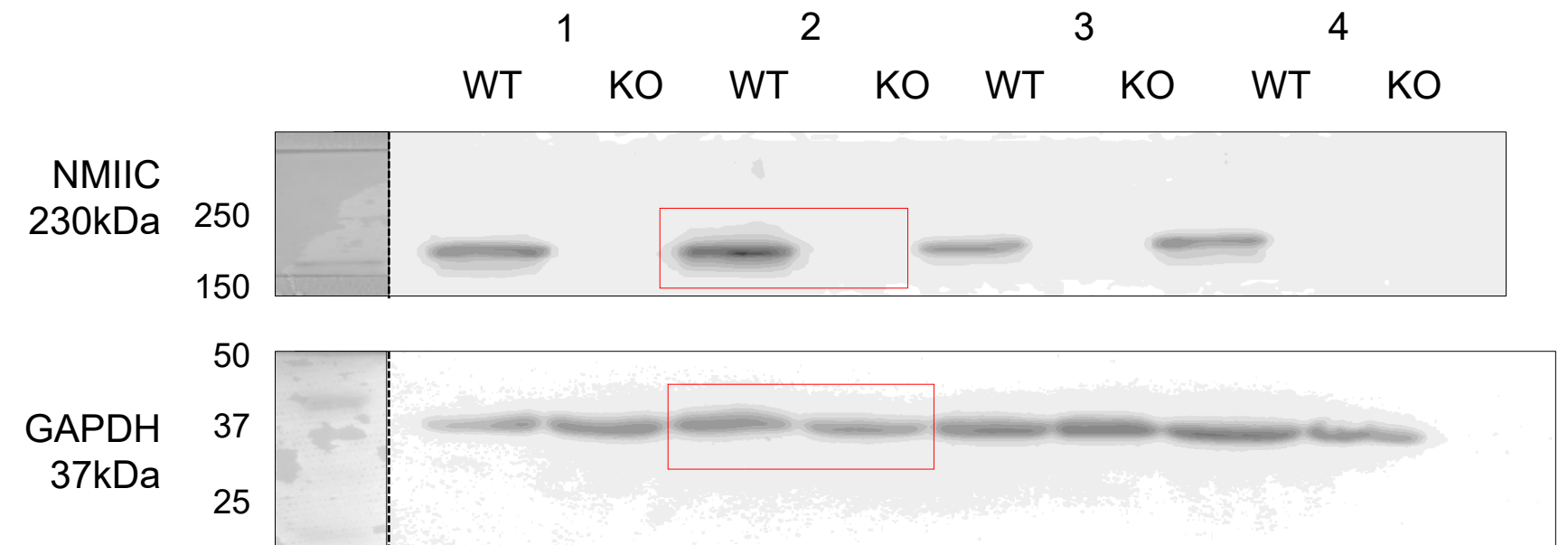

Fig 6a

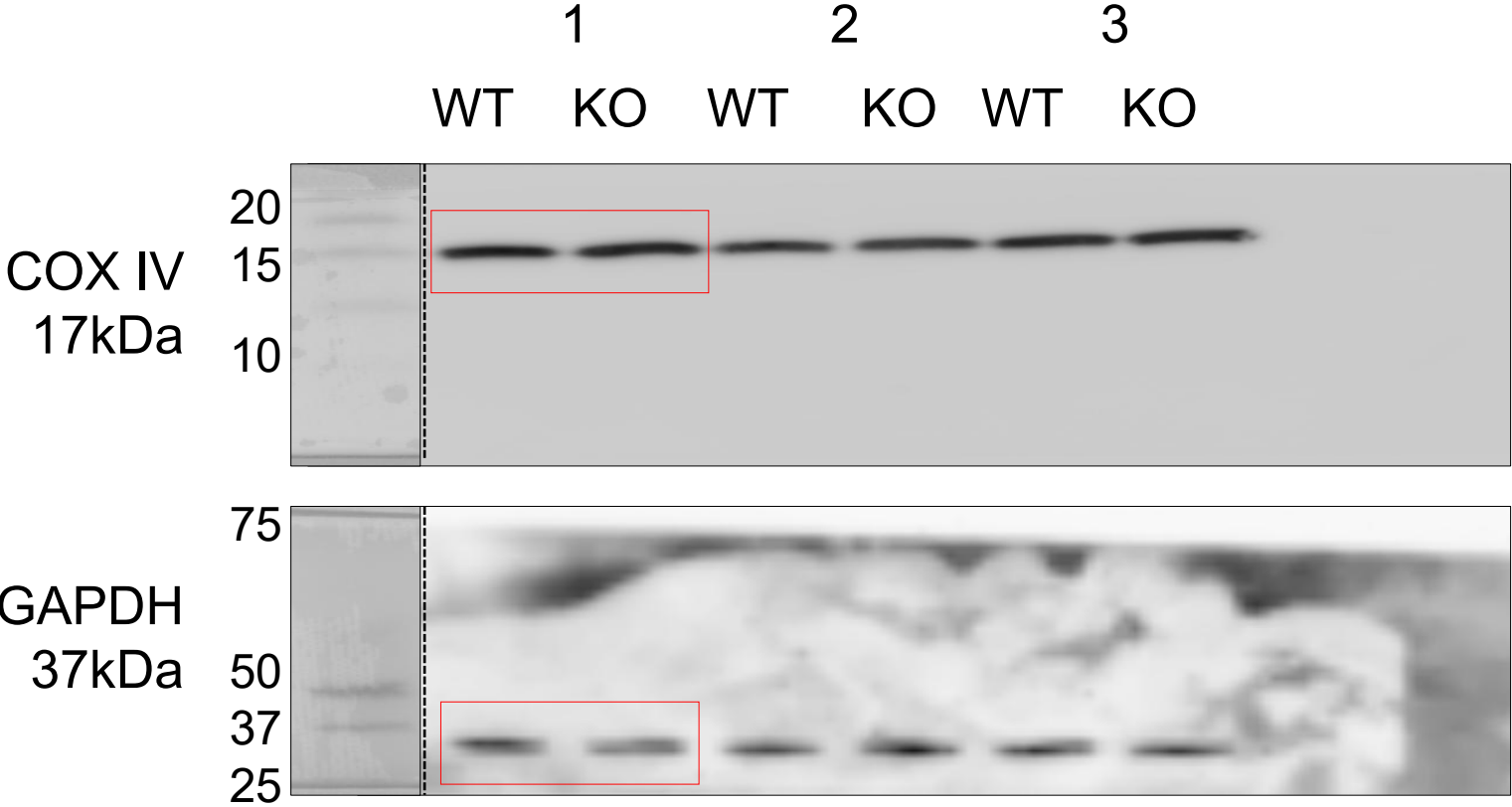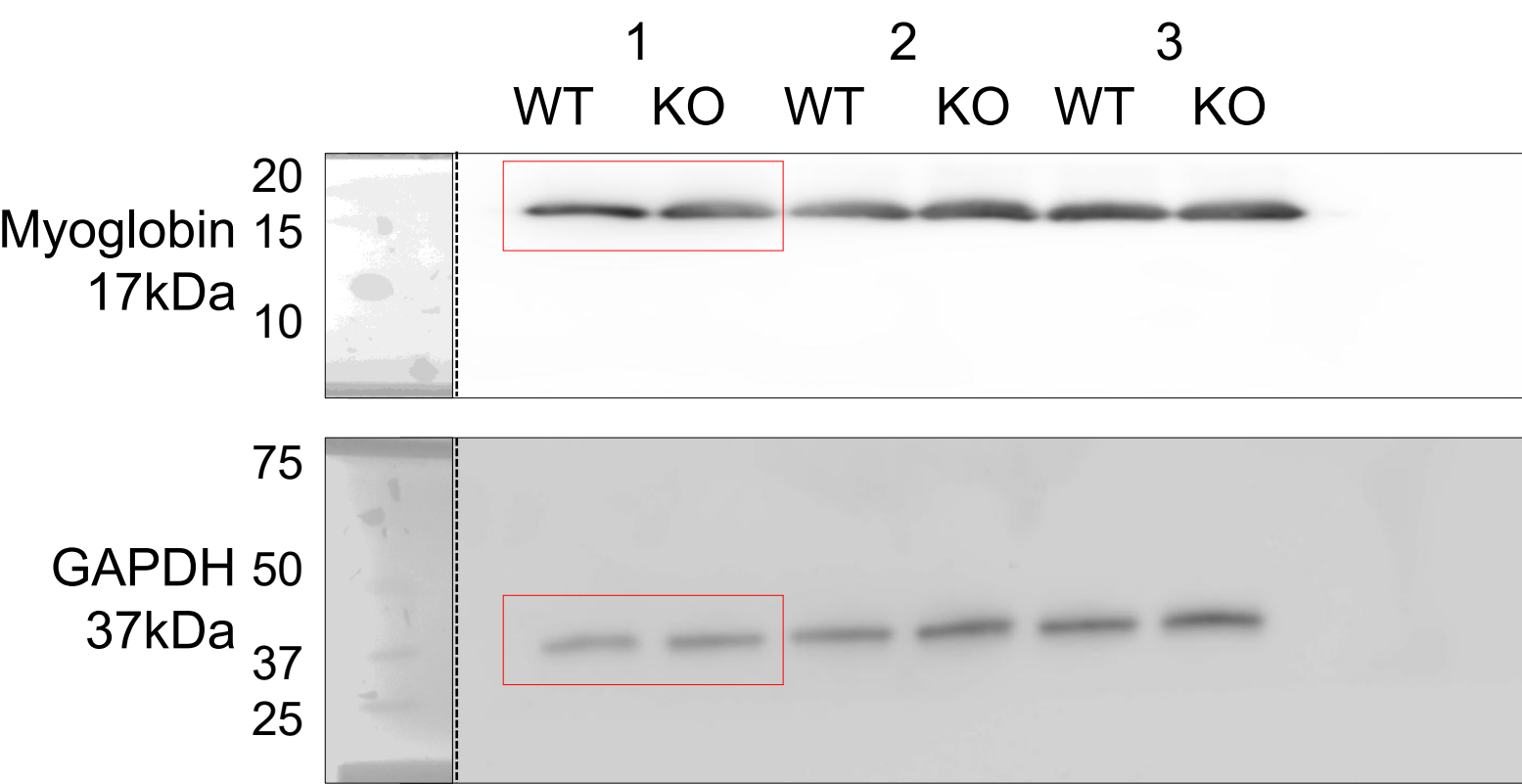

Fig 7a

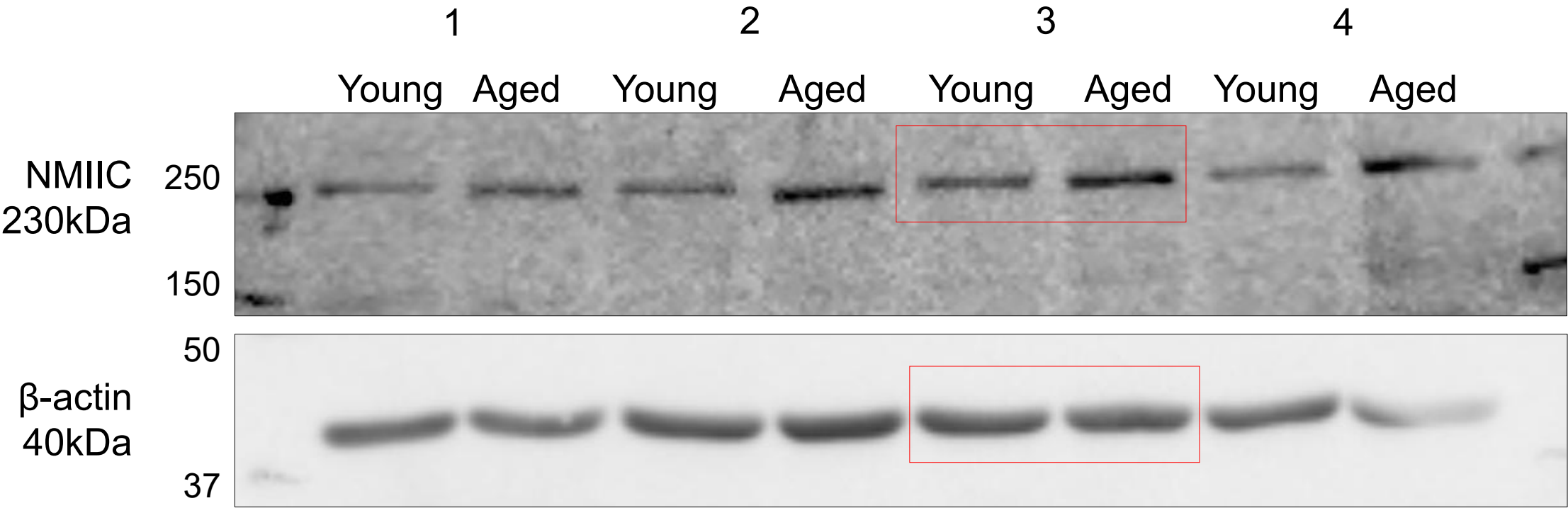

S1a Fig

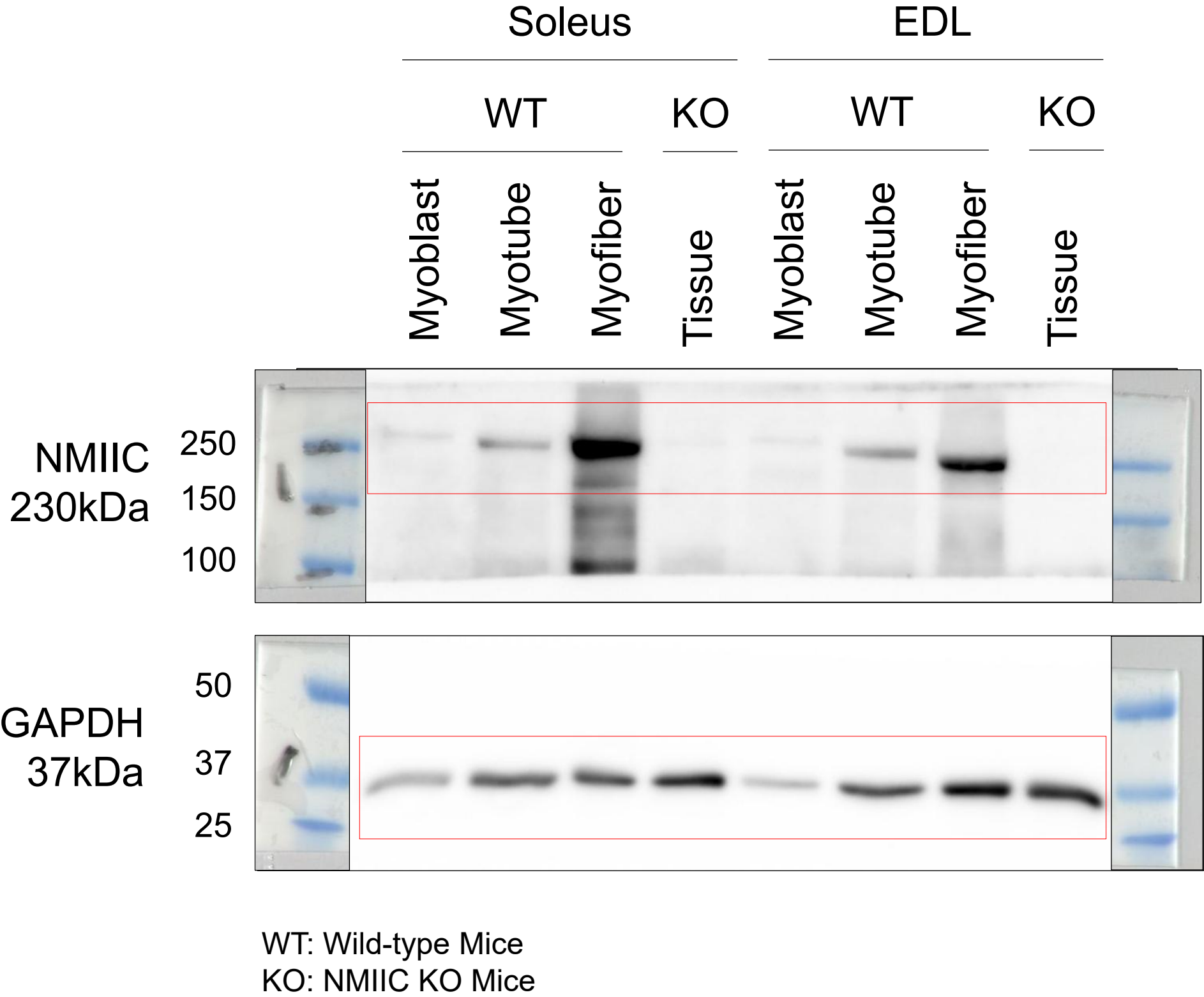

S1b Fig

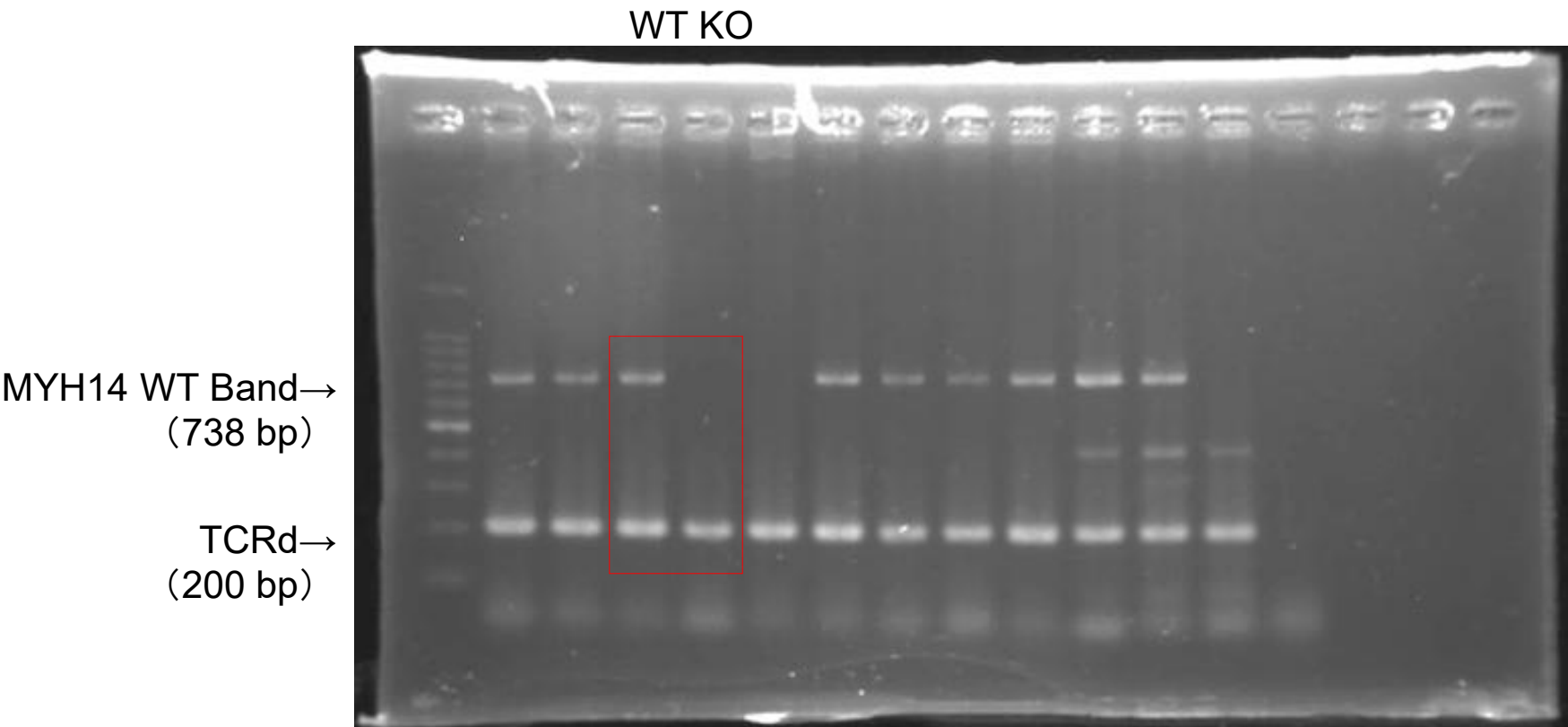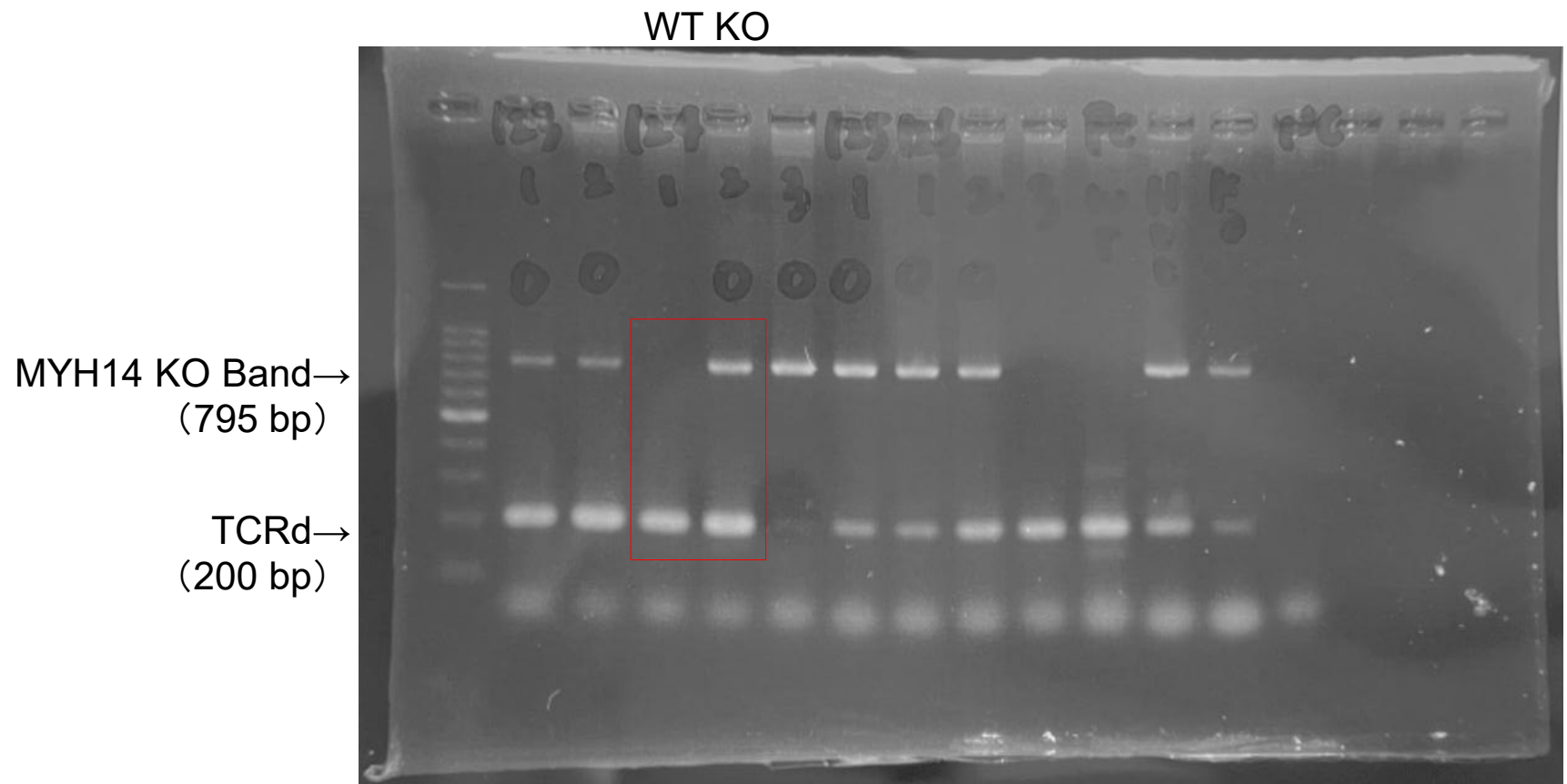

Supplement: S1 Raw Images — (PDF) [file pone.0337708.s004.pdf]
